# The kinetoplastid-specific transcription factor mtTF1 is essential for mitochondrial gene expression and maxicircle replication in *Trypanosoma brucei*

**DOI:** 10.1371/journal.pbio.3003945

**Published:** 2026-09-21

**Authors:** Bianca Manuela Berger, Enrico Santini, Himaja Manjunatha, Lea Trost, Markus Gerber, Hauke S. Hillen, Torsten Ochsenreiter

**Affiliations:** 1 Institute of Cell Biology, University of Bern, Bern, Switzerland; 2 Graduate School for Cellular and Biomedical Sciences, University of Bern, Bern, Switzerland; 3 Department of Cellular Biochemistry, University Medical Center Göttingen, Göttingen, Germany; 4 Research Group Structure and Function of Molecular Machines, Max Planck Institute for Multidisciplinary Sciences, Göttingen, Germany; University of Melbourne, AUSTRALIA

## Abstract

Mitochondrial DNA replication and gene expression are essential for cell survival. In *Trypanosoma brucei*, a protozoan animal and human parasite, the mitochondrial RNA polymerase (mtRNAP, Tb927.11.5780) plays roles in both transcription and DNA replication. This study identifies and characterizes the first mitochondrial transcription factor (mtTF1, Tb927.6.4510) in the Kinetoplastea. The mtRNAP and mtTF1 form a high-molecular-weight complex that localizes to the kinetoplast DNA (kDNA) and is essential for parasite survival in both life cycle stages. Their localization is interdependent, and both proteins influence maxicircle replication, but not minicircle replication. Knockdown of either protein results in altered gene expression, particularly affecting the minor strand of the mitochondrial genome. mTF1 specifically binds to kDNA and since it is unique to the Kinetoplastea, it might prove to be a promising drug target.

## Abstract 2

How mitochondrial transcription and DNA replication are coordinated in Trypanosoma brucei remains unclear. This study identifies mtTF1, the first mitochondrial transcription factor in Kinetoplastea required for gene expression and maxicircle replication.

## Introduction

Mitochondria are membrane-bound organelles found in nearly all eukaryotic cells. Often referred to as the “powerhouse of the cell”, they generate energy in the form of ATP through oxidative phosphorylation, rendering them important for cell survival and viability [1]. Consistent with the endosymbiont theory, mitochondria possess their own DNA, reflecting their bacterial ancestry [2]. However, over the course of evolution, the mitochondrial genome was reduced, and some genes were transferred to the nucleus [2]. Consequently, many mitochondrial proteins require import into the mitochondria [3]. In contrast to nuclear gene expression, mitochondrial gene expression relies on a distinct set of proteins, most of which are encoded in the nucleus [4,5]. According to the endosymbiont theory, a eubacterial-like multisubunit RNA polymerase would be expected in mitochondria. However, the mitochondrial RNA polymerase (mtRNAP, also known as POLRMT), the single-subunit enzyme responsible for transcribing mitochondrial DNA (mtDNA) into RNA, shares structural and functional similarities with the T7 phage RNA polymerases and is conserved across most eukaryotes [6]. While T7 phage RNA polymerases transcribe genes in a transcription factor-independent manner, all known eukaryotic mtRNAPs require transcription factors for their function [7]. These transcription factors vary significantly across species. In human mitochondria, for instance, transcription involves four transcription factors [5,8]. Two of which are involved in transcription initiation; mitochondrial transcription factor A (TFAM) binds and recruits mtRNAP to promoters, while transcription factor B2 (TFB2M) helps to open the DNA [9–11]. Another factor, mitochondrial transcription elongation factor (TEFM), replaces TFB2M and enhances the processivity of mtRNAP, thereby promoting the elongation of RNA transcripts [10]. Transcription initiated at the light strand promoter (LSP) is terminated by the mitochondrial transcription termination factor (mTERF), which acts as a roadblock [12]. In contrast to human mitochondrial transcription, yeast mitochondrial transcription requires only one mitochondrial transcription factor (Mtf1), which is homologous to TFB2M found in humans [11,13–15]. Transcription factors are much less conserved across eukaryotes compared to the mtRNAP, with considerable variation in their functional role structures, regulatory mechanisms.

Besides mitochondrial transcription, mtRNAP is also involved in primer synthesis for mtDNA replication [5]. Interestingly, transcription from the LSP can be prematurely terminated, with the resulting short RNA acting as a primer for replication from the heavy strand origin of replication (OriH) [9]. Once the replication machinery completed two-thirds of the heavy-strand replication, the light-strand origin (OriL) becomes single-stranded and forms a stem-loop structure, which is accessible to mtRNAP. The mtRNAP then synthesizes the primer at OriL, which is subsequently used for light-strand replication [16–18]. In this strand-displacement model, which is the currently most-accepted model, no Okazaki fragments are required for lagging strand synthesis in humans [19]. Importantly, mtRNAP regulates the switch between primer formation and gene expression [20]. Heterozygous knockout experiments revealed that under low mtRNAP levels, transcription at the LSP is favored, ensuring primer formation and, consequently, a preference for replication over transcription [5,20].

*Trypanosoma brucei* is the parasitic protozoan responsible for the diseases African trypanosomiasis (sleeping sickness) in humans and Nagana in cattle. Its metabolism strategies are tailored to different environments, allowing the parasite to grow in the mammalian host or the tsetse fly vector [21,22]. These metabolic adaptations are essential for survival under diverse physiological conditions. In the mammalian bloodstream, the bloodstream form (BSF) trypanosomes rely almost exclusively on glycolysis for ATP production as glucose is abundant. In this stage, the single mitochondrion is reduced, and oxidative phosphorylation is downregulated [23]. In contrast, in the tsetse fly vector, trypanosomes exist as procyclic form (PCF), which has fully developed and functional mitochondria that allow them to use oxidative phosphorylation for ATP generation via the electron transport chain [23,24]. The mechanisms underlying this metabolic plasticity are not yet fully understood. Hypotheses include the involvement of life cycle-specific transcription factors, post-transcriptional mechanisms such as editing or processing, as well as mRNA degradation and substrate availability [25,26].

An additional layer of complexity comes through the complex mitochondrial DNA network found in trypanosomes, which is composed of two different types of circular DNA molecules [27]. There are ~25 maxicircles (23 kb in size), which encode proteins of the respiratory chain, two rRNAs, and two ribosomal proteins [25,28]. These maxicircles are similar to other eukaryotic mtDNA molecules, with the major difference being that no tRNAs are encoded in the mitochondrial DNA of trypanosomes, rendering them dependent on tRNA import into the mitochondrion [29,30]. In addition to maxicircles, *T. brucei* contains approximately 5,000 minicircles per cell, each around 1 kb in size. The minicircles encode for 3–5 gRNAs required for mRNA editing of maxicircle-encoded transcripts [31,32]. Interestingly, it has been shown that mtRNAP (Tb927.11.5780) not only transcribes maxicircles, but that the protein is also involved in transcription of gRNAs encoded on minicircles [33,34]. It is still unknown whether gene expression of these two DNA molecules is regulated by a common or different transcription factors.

In the present study, immunoprecipitation experiments of the mtRNAP led to the identification of the first mitochondrial transcription factor (mtTF1, Tb927.6.4510) in *T. brucei.* Further experiments confirmed the co-localization of mtRNAP and mtTF1 at the kDNA and demonstrated their interdependence. We show that mtTF1 is involved in both maxicircle and minicircle transcription. Upon mtTF1 knockdown, we observed strand-specific effects on maxicircle transcription, with the minor strand transcripts showing a more significant decrease compared to major strand transcripts. A similar observation was made upon ablation of mtRNAP. It remains to be elucidated whether the promoters for major and minor strand transcription have different strengths, accounting for this phenotype. Additionally, both mtRNAP and mtTF1 are required for maxicircle but not minicircle replication. Interestingly, mtTF1 is conserved among all Kinetoplastea, including human pathogens such as *Leishmania* and *Trypanosoma cruzi*. Given that mtTF1 is essential in *T. brucei*, it may represent an interesting candidate for drug development.

## Results

### The mitochondrial RNA-polymerase (mtRNAP) localizes to the kDNA and is essential in the procyclic and bloodstream form trypanosomes

The mitochondrial RNA-polymerase (mtRNAP, Tb927.11.5780) of *T. brucei* has been identified by Grams and coworkers in 2002 [33]. The authors showed the involvement of mtRNAP in maxicircle replication, maxicircle transcription and the essentiality of the protein for the PCF of *T. brucei* [33]. Additionally, Hashimi and coworkers could show that mtRNAP is also involved in minicircle transcription [34]. To further investigate the role of mtRNAP, we first C-terminally tagged mtRNAP with a 3xMyc tag and determined the localization of the protein. Biochemical analysis using digitonin fractionation showed that mtRNAP-Myc localizes to the mitochondria-enriched fraction (Fig 1A). The tagged mtRNAP-Myc is found in the mitochondria-enriched pellet, whereas no signal was detected in the supernatant containing the cytosolic proteins (Fig 1A). With immunofluorescence analysis (IFA), we observed mtRNAP-Myc localization at the kDNA throughout the cell cycle in the PCF (Fig 1B). Next, inducible mtRNAP RNAi cell lines were generated in PCF and BSF trypanosomes. The RNAi effectively resulted in depletion of the protein as shown by western blot (Fig 1C and 1D inlets). RNAi depletion of mtRNAP results in loss of fitness starting after 2–3 days of induction in the PCF (Fig 1C). Since the PCF and BSF have differential dependence on mitochondrial oxidative phosphorylation for ATP production, we wanted to investigate if mtRNAP is essential in the BSF as well. Depletion of mtRNAP in BSF results in a growth phenotype starting after two to three days of induction, indicating that mtRNAP is essential in both the PCF and BSF of *T. brucei* (Fig 1D). The functionality of the tagged version of mtRNAP was tested and while the protein was found to localize correctly, it was unable to rescue the RNAi based knockdown of the endogenous mtRNAP (S1 Fig).

**Fig 1:**
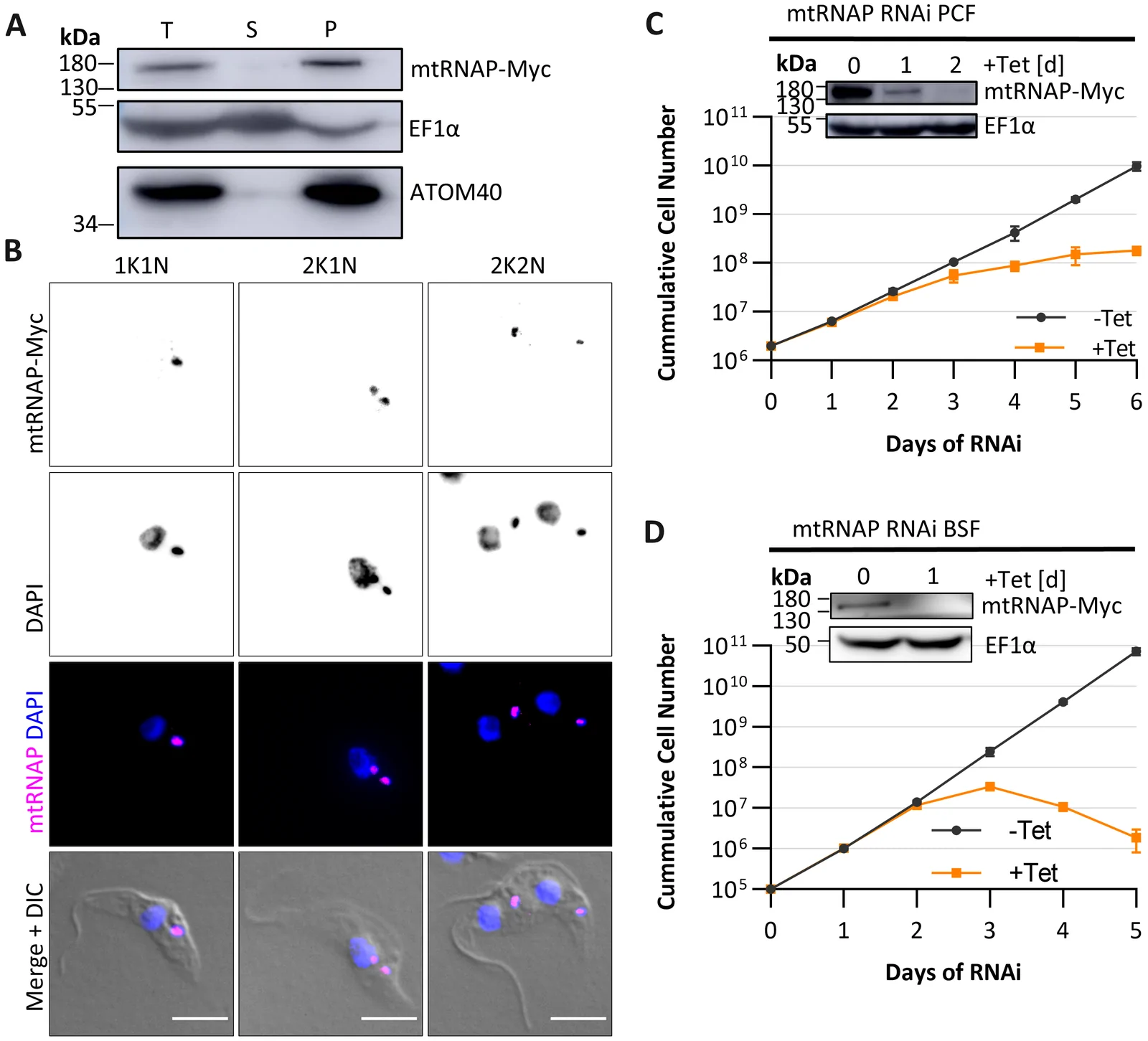
mtRNAP localizes to the kDNA and is essential in the procyclic form (PCF) and bloodstream form (BSF) of trypanosomes. **(A)** western blot of total cell lysate (T), the soluble cytosolic fraction (S), and the digitonin-extracted mitochondria-enriched pellet (P) of cells expressing C-terminally Myc-tagged mtRNAP. The western blot was probed for EF1α and ATOM40, which serve as cytosolic and mitochondrial markers, respectively. **(B)** Immunofluorescence images showing localization of the Myc-tagged mtRNAP (magenta) at the kDNA throughout the cell cycle (K = kDNA, N = nucleus). DNA is stained with DAPI (blue). Cells are visualized in DIC (differential interference contrast microscopy). Additionally, inverted black and white images are shown for better visualization. **(C** and **D)** Growth curve of noninduced (black, -Tet) and mtRNAP RNAi-induced (gray, +Tet) cells in the procyclic form (PCF) and bloodstream form (BSF), respectively. Inset: western blot of noninduced and mtRNAP RNAi-induced cells (+Tet). Western Blot was probed for Myc to detect mtRNAP levels and EF1α, which served as a loading control. Scale bars in B = 5 µm. Data are shown as mean +/− standard deviation (SD). *n* = 3 for C and D. Statistical significance was determined using an unpaired two-tailed Student *t* test. *p* < 0.05 (*), *p* < 0.01 (**), *p* < 0.001 (***). The data underlying this Figure can be found in S1 Data.

### Immunoprecipitation of mtRNAP to identify interaction partners of mtRNAP in *T. brucei*

To identify interaction partners of mtRNAP in *T. brucei*, we performed immunoprecipitation with mtRNAP tagged C-terminally with an HA-tag (PCF) or myc-tag (BSF). For the immunoprecipitation, cells were lysed, and mitochondria were enriched using digitonin fractionation. The workflow is shown in S2 Fig. The input, flowthrough and immunoprecipitation fractions were analyzed by western blot (Fig 2A). The mtRNAP-HA is detected in the input, as well as the immunoprecipitation fraction, whereas the outer mitochondrial membrane protein ATOM40 (atypical translocase of the mitochondrial outer membrane 40) is found in the input and flowthrough (Fig 2A). Mass spectrometry revealed that mtRNAP and the hypothetical protein Tb927.6.4510 (hereafter called mitochondrial transcription factor 1: mtTF1) were the most enriched proteins in the mtRNAP-HA pulldown experiments in PCF as well as BSF trypanosomes (Fig 2B and 2C). Tables 1 and 2 summarize the significantly enriched proteins in the mtRNAP immunoprecipitation performed in PCF and BSF, respectively, with a fold change larger than two (S1 and S2 Tables). We confirmed the interaction between the endogenous mtRNAP and a tagged version of mtTF1. In this additional experiment the endogenous mtRNAP was the most enriched protein after mtTF1-myc immunoprecipitation and mass spectrometry (S3 Fig).

**Table 1:** Significantly enriched proteins in the mt*RNAP* immunoprecipitation in the *PCF*. Shown are hits with a fold change larger than two.

Rank of log2 FC	Accession number	Protein	Location based on TrypTag [35] (C-terminal tagging)	HyperLOPIT localization (PCF) [36]
1	Tb927.11.5780	mtRNAP	Cytoplasm, mitochondrion	Mitochondrion- Matrix 2
2	Tb927.6.4510	hypothetical protein (mtTF1)	Kinetoplast	Mitochondrion- Matrix 2
3	Tb927.9.7670	hypothetical protein	Nucleolus	Nucleus
4	Tb927.11.15850	mitoribosomal SSU assembly factor 5	Cytoplasm, mitochondrion	Unknown
5	Tb927.11.6980	phosphoenolpyruvate carboxykinase	Mitochondrion, kinetoplast	Mitochondrion- Matrix 2
6	Tb927.9.1980	hypothetical protein	Axoneme, cytoplasm	Flagellum 1
7	Tb927.11.3120	nucleolar GTP-binding protein 1	Nucleolus	Nucleus

**Table 2:** Significantly enriched proteins in the mt*RNAP* immunoprecipitation in the *BSF*. Shown are hits with a fold change larger than two.

Rank of log2 FC	Accession number	Protein	Location based on TrypTag [35](C-terminal tagging)	HyperLOPIT localization (PCF) [36]
1	Tb927.6.4510	hypothetical protein (mtTF1)	Kinetoplast	Mitochondrion- Matrix 2
2	Tb927.11.5780	mtRNAP	Cytoplasm, mitochondrion	Mitochondrion- Matrix 2
3	Tb927.9.4210	fatty acyl CoA synthetase 3	–	Secretory/endocytic 2
4	Tb927.10.5770	valosin-containing protein	Nucleoplasm, cytoplasm	Cytosol
5	Tb927.5.1300	V-type proton ATPase subunit a	Endocytic	Secretory/endocytic 2

**Fig 2:**
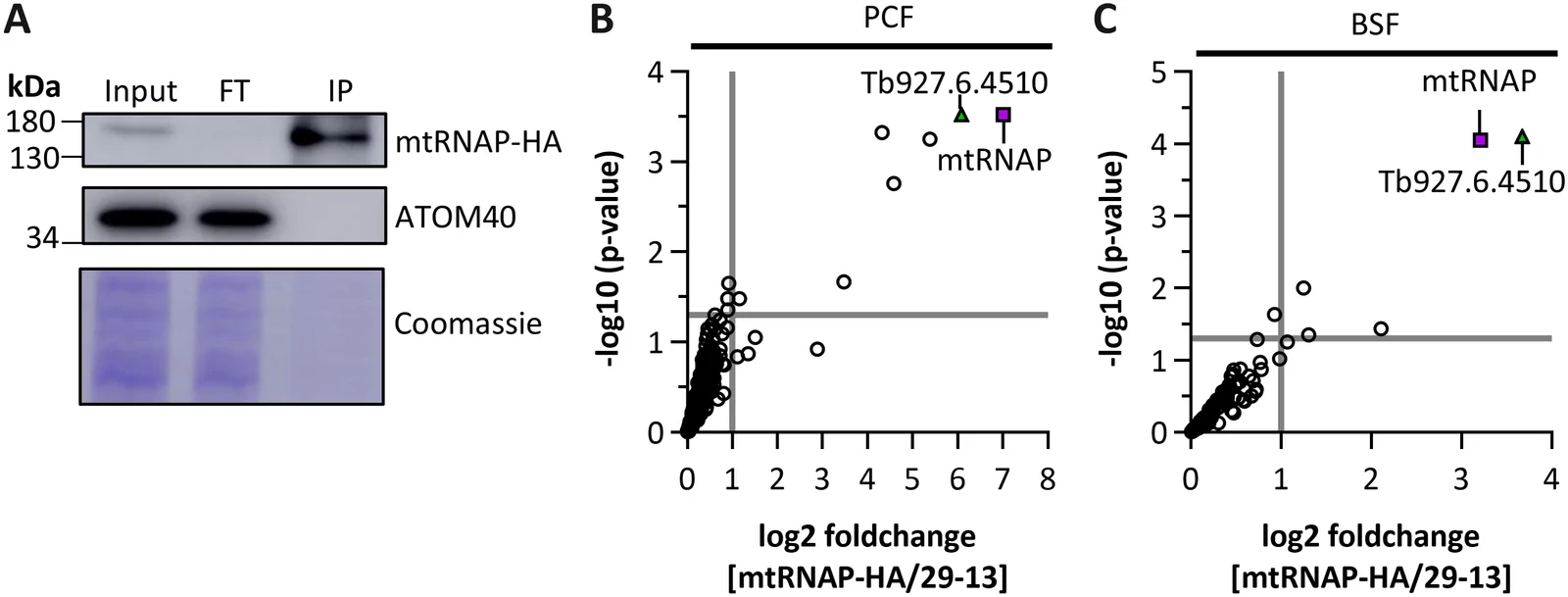
Immunoprecipitation analysis of mtRNAP to identify interaction partners. **(A)** western blot analysis of the immunoprecipitation performed with PCF cells expressing HA-tagged mtRNAP. Lysed mitochondria were used as input for the immunoprecipitation. The mitochondrial protein ATOM40 serves as a control and is expected in the flowthrough (FT), while mtRNAP is expected in the immunoprecipitation fraction (IP). **(B** and **C)** Volcano plot of the immunoprecipitation of cells expressing the tagged mtRNAP in the procyclic form (PCF) and the bloodstream form (BSF), respectively. Wild-type cells without tagged mtRNAP served as negative control for background subtraction. Horizontal lines indicate a *p*-value of 0.05 and vertical lines mark a 2-fold enrichment of proteins in the tagged cell lines compared to wildtype cells. *n* = 3 for B and C.

### mtTF1 is conserved in the Kinetoplastea and co-localizes with mtRNAP at the kDNA

The protein mtTF1 is 37 kDa in size with an isoelectric point (IP) of 6.9, which is in a similar range to the IP of mtRNAP (IP = 7.07) [37]. mtTF1 is conserved in the Kinetoplastea but not found in other eukaryotic organisms (Fig 3A). AlphaFold3 was used to predict the structure of mtTF1 (Fig 3B) [38]. The N-terminal domain of the mtTF1 prediction has a lower confidence score and potentially contains three alpha helices. The middle part of the predicted protein structure until the C-terminus, is composed of helix-turn-helix repeats, predicted with high confidence (Fig 3B). However, mtTF1 has no recognizable domains predicted. To verify the interaction between mtRNAP and mtTF1, a double-tagged cell line was generated in the PCF: mtRNAP-HA and mtTF1-Myc. Co-immunoprecipitation with beads coupled to anti-HA antibodies (pulldown of mtRNAP-HA) showed that mtRNAP-HA as well as mtTF1-Myc were in the co-immunoprecipitation fraction (Fig 3C). Similar results were obtained performing reciprocal co-immunoprecipitation with beads coupled to anti-Myc antibodies (pulldown of mtTF1-Myc) (Fig 3D). Importantly, samples for co-immunoprecipitation were treated with RNase and DNase, indicating that the interaction between mtRNAP and mtTF1 is RNA- and DNA-independent (Fig 3C and 3D). IFA of mtRNAP and mtTF1 showed that the two epitope-tagged proteins colocalize at the kDNA throughout the cell cycle (Fig 3E) in the PCF. For better resolution of mtRNAP and mtTF1 and to calculate the Pearson correlation coefficient, we performed Ultrastructure Expansion Microscopy (U-ExM) with the Pre-Staining Expansion Microscopy (PS-ExM) protocol developed by Atchou and coworkers [39]. Signal of mtRNAP and mtTF1 overlap at the kDNA disk in the PCF with a Pearson correlation coefficient of 0.80 ± 0.07 (the average ± SEM of 10 kDNAs was calculated with Fisher’s transformation).

**Fig 3:**
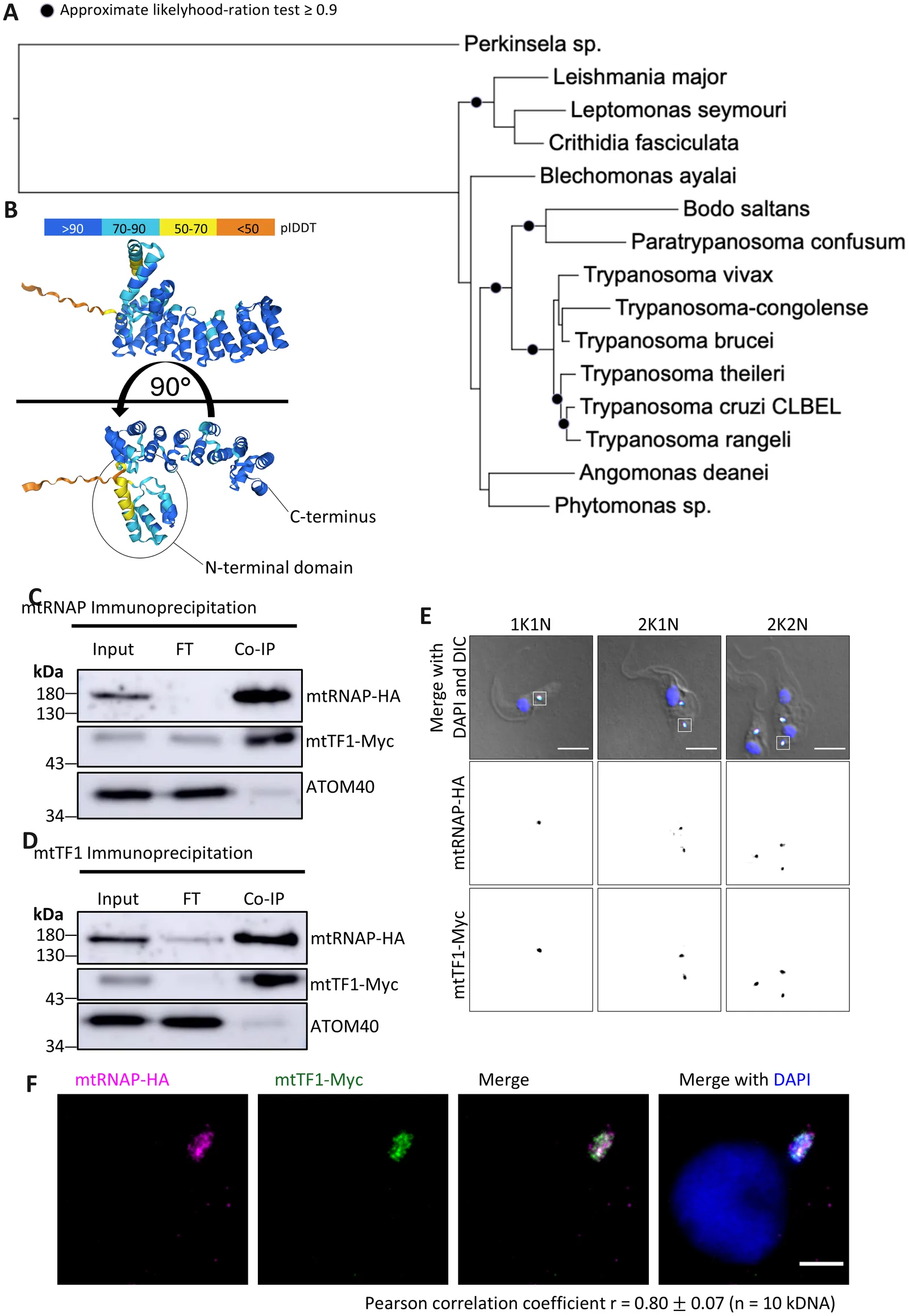
mtTF1 is conserved in the Kinetoplastea and co-localizes with mtRNAP at the kDNA in the PCF. **(A)** Phylogenetic tree illustrating the conservation of mtTF1 among the Kinetoplastea. **(B)** AlphaFold3 prediction of mtTF1. The N-terminal domain is marked with a circle. **(C** and **D)** western blot of the co-immunoprecipitation (Co-IP) performed with cells expressing the HA-tagged mtRNAP and Myc-tagged mtTF1 using anti-HA and anti-Myc beads, respectively. Digitonin-extracted mitochondria were used as input for the Co-IP. Western blots were probed with anti-HA, anti-Myc, and anti-ATOM40 antibodies. ATOM40 serves as a negative control, which is not expected in the Co-IP fraction, but only in the flowthrough (FT). **(E)** Immunofluorescence images showing the localization of mtRNAP-HA (magenta) and mtTF1-Myc (green) throughout the cell cycle (K = kDNA, N = nucleus). DNA is visualized with DAPI (blue) and cells are visualized in DIC (differential interference contrast microscopy). Additionally, inverted black and white images are shown for better visualization. **(F)** Expansion microscopy images of cells expressing mtRNAP-HA (magenta) and mtTF1-Myc (green). DNA is stained with DAPI (blue). Pearson correlation coefficient in panel F was calculated for n = 10 kDNA. Data are presented as mean +/− SEM. Scale bars = 5 µm. The data underlying this Figure can be found in S1 Data.

### mtTF1 is essential in the PCF and is in a complex with the mtRNAP

Conditional knockdown of mtTF1 was performed by inducible RNAi, which results in depletion of the protein as verified with western blot using the mitochondrial heat shock protein 70 (mtHSP70) as a loading control (Fig 4A). We observed a decrease in protein levels of the nuclear-encoded mitochondrial cytochrome c oxidase subunit 4 (COX4) after 1 day of mtTF1 RNAi induction, and thus prior to the general loss of fitness starting on day four. Loss of fitness is observed starting after 3–4 days of mtTF1 RNAi induction (Fig 4B). To test if the proper localization of mtTF1 at the kDNA depends on the presence of mtRNAP, we generated a cell line with an HA-epitope tagged mtTF1 (C-terminal tagging) and inducible mtRNAP RNAi. The growth curve is shown in S4A Fig. Two days after induction of mtRNAP knockdown, mtTF1 largely lost its localization to the kDNA (Fig 4C and 4D), while it remained detectable in the mitochondrial fraction (Fig 4E). These findings were quantified by measuring the signal of HA-tagged mtTF1 at the kDNA and next to the kDNA in control (−Tet) and mtRNAP depleted cells (+Tet [2d]) (Fig 4D). While the signal intensity of mtTF1-HA at the kDNA decreased upon mtRNAP RNAi, the signal intensity of mtTF1-HA next to the kDNA increased (Fig 4D). To verify if mtTF1 still localizes to the mitochondrion upon mtRNAP depletion, we performed digitonin extraction. The mtTF1-HA was detected in the mitochondria-enriched fraction in the control cells and upon mtRNAP depletion (Fig 4E). Blue-Native PAGE confirmed that mtRNAP-HA and mtTF1-HA are in a high-molecular weight complex of similar size (S2B Fig). Next, we tested the integrity of the high-molecular weight complex upon knockdown of mtRNAP. In the tested condition, mtRNAP depletion leads to loss of mtTF1-HA within the high-molecular weight complex (Fig 4F). Western blot of a duplicate experiment revealed no effect on mtTF1-HA levels upon mtRNAP RNAi 2 days after induction (Fig 4F). Thus, we conclude that (i) the localization of mtTF1 to the kDNA depends on mtRNAP and (ii) the formation of the mtTF1-containing high-molecular weight complex requires the presence of the mtRNAP. To test the interaction of the two proteins in vitro, we co-expressed mtTF1 and a N-terminally truncated version of the mitochondrial RNA polymerase with a N-terminal maltose binding protein in insect cells. After cell lysis and purification using amylose resin the bound mtRNAP was released by TEV protease cleavage. The eluate was fractionated by size exclusion chromatography and the peak fractions analyzed by SDS PAGE (S8A Fig). In the peak fractions both the mtRNAP and the co-purified mtTF1 could be identified and verified by mass spectrometry (S8B Fig). In summary, mtTF1 is essential for procyclic-form viability and respiratory chain maintenance, and its localization to the kinetoplast DNA as well as its incorporation into a high-molecular-weight mitochondrial complex strictly depend on the presence of the mitochondrial mtRNAP, with which it forms a direct physical interaction in vitro and in vivo.

**Fig 4:**
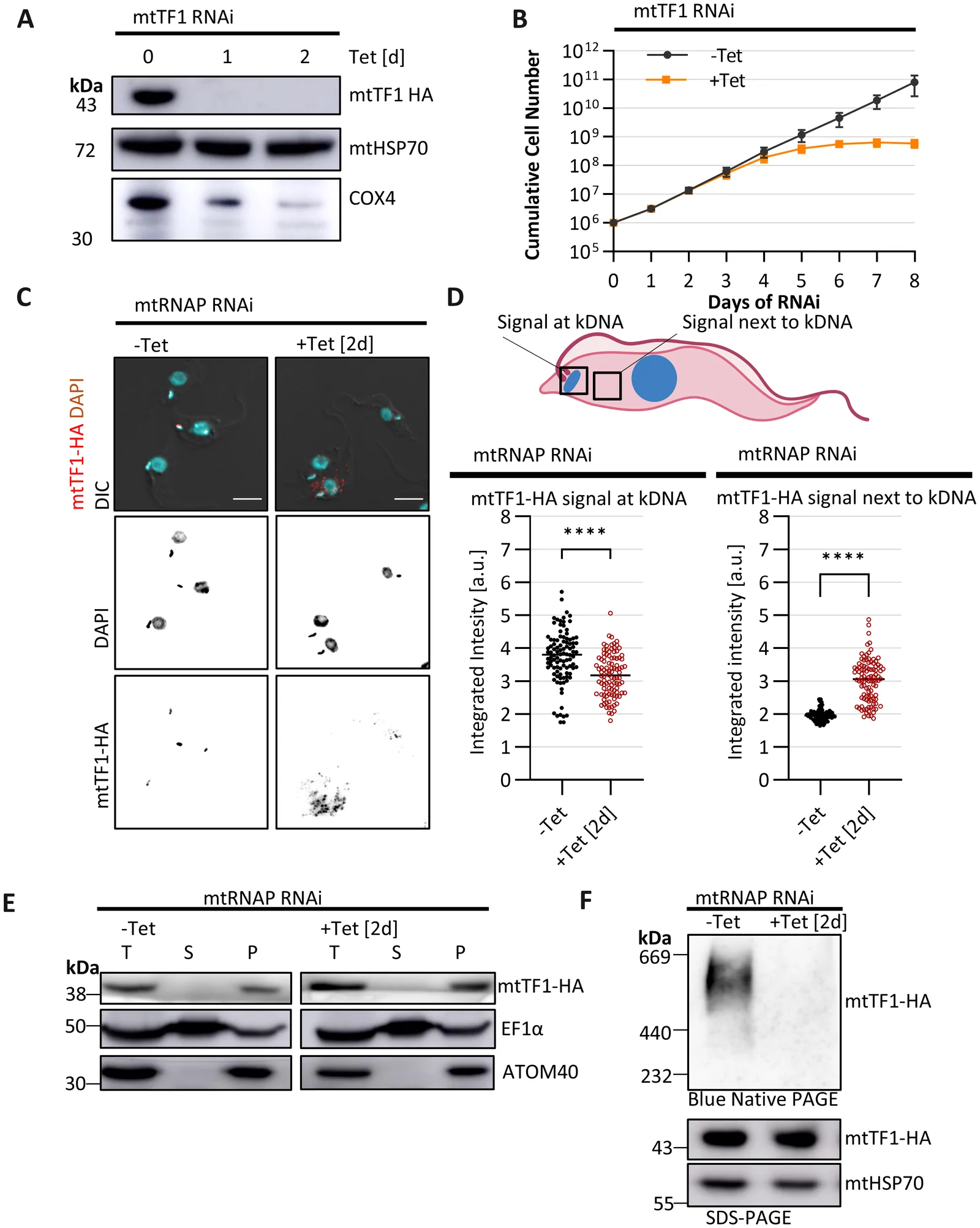
mtTF1 is essential in the insect form, and its localization at the kDNA depends on mtRNAP. **(A)** western blot analysis showing depletion of mtTF1 after 1 and 2 days of tetracycline-induced mtTF1 RNAi compared to noninduced control cells. The western blot was probed for COX4 to assess the integrity of mitochondrial respiratory chain complexes, while mtHSP70 serves as a loading control. **(B)** Growth curve of PCF trypanosomes, upon tetracycline-induced mtTF1 RNAi (+Tet) cells compared to noninduced control cells (−Tet). **(C)** Immunofluorescence images showing mtTF1-HA localization (red) in control cells (−Tet) and upon induction of mtRNAP RNAi (+Tet[2d]). DNA is stained with DAPI (cyan). Cells are visualized with DIC (differential interference contrast microscopy). **(D)** Quantification of mtTF1-HA signal intensity at the kDNA (left) and next to the kDNA (right) upon mtRNAP RNAi (+Tet [2d], red) compared to noninduced control cells (−Tet, black). **(E)** Digitonin extraction was performed in control cells (−Tet) and mtRNAP RNAi-induced cells (+Tet [2d]) to biochemically investigate the localization of mtTF1 upon mtRNAP depletion. western blot analysis of total cell lysate (T), the soluble cytosolic fraction (S), and digitonin-extracted mitochondria-enriched pellet (P). The western blot was probed for HA to detect mtTF1. EF1α and ATOM40 serve as cytosolic and mitochondrial marker, respectively. **(F)** Blue Native PAGE analysis (top) of mtTF1-HA in mtRNAP RNAi-induced cells (+Tet [2d]) and noninduced controls (−Tet). Western blot (bottom) probed for HA to detect mtTF1 upon mtRNAP depletion (+Tet [2d]) compared to noninduced controls (−Tet). Blue Native PAGE and SDS-PAGE were performed as duplicate experiments. Scale bars = 5 µm. Data are shown as mean +/− standard deviation (SD). *n* = 3 for B and F. *n* = 100 for E. Statistical significance was determined using an unpaired two-tailed Student *t* test. *p* < 0.05 (*), *p* < 0.01 (**), *p* < 0.001 (***). The data underlying this Figure can be found in S1 Data.

### mtRNAP localization to the kDNA depends on mtTF1

Knockdown of mtTF1 was used to investigate how the localization of mtRNAP and the high-molecular weight complex containing mtRNAP depend on the novel identified factor. The growth curve is shown in S4C Fig. When mtTF1 is depleted, mtRNAP intensity at the kDNA is reduced (Fig 5A and 5B), while the signal of mtRNAP next to the kDNA is not affected by mtTF1 RNAi (Fig 5C). Next, we performed Blue Native PAGE to test whether mtTF1 depletion affects the high-molecular weight complex. We observed a decrease in the size of the complex when mtTF1 is depleted, indicating loss of mtTF1 from the complex (Figs 5D and S2D). mtRNAP-HA levels were not affected by mtTF1 knockdown as assessed by western blot analysis (Fig 5E). These results show that mtRNAP is still present in a high-molecular weight complex upon mtTF1 RNAi. Similar to the observation that mtTF1 localization depends on mtRNAP, the localization of mtRNAP at the kDNA strongly depends on mtTF1.

**Fig 5:**
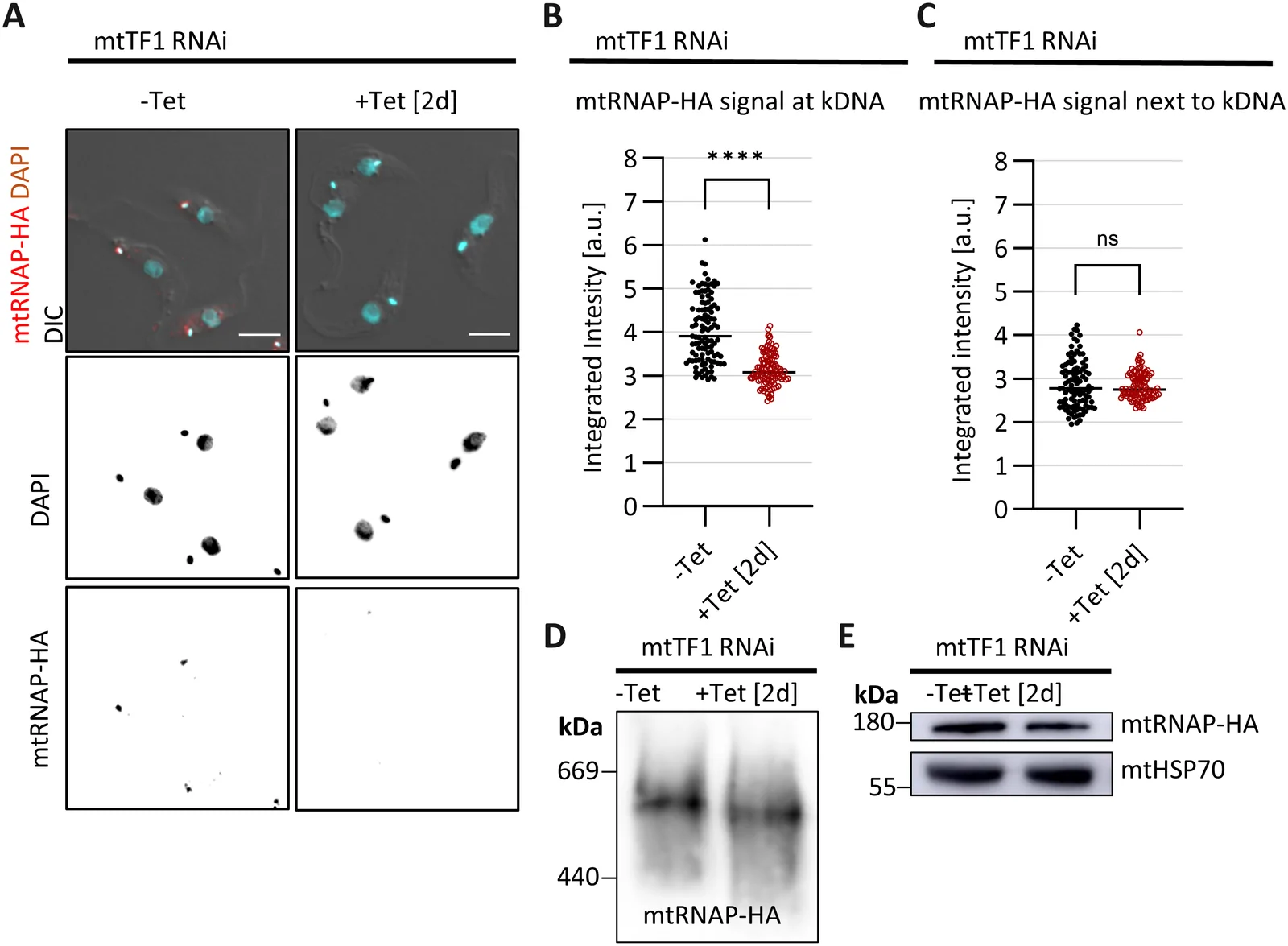
mtRNAP intensity at the kDNA decreases upon mtTF1 RNAi in the PCF. **(A)** Immunofluorescence images showing mtRNAP-HA localization (red) upon mtTF1 RNAi. DNA is stained with DAPI (cyan). Cells are visualized with DIC (differential interference contrast microscopy). **(B** and **C)** Quantification of the mtRNAP-HA signal intensity assessed by immunofluorescence imaging upon mtTF1 knockdown (+Tet [2d], red) compared to noninduced control cells (−Tet, black). mtRNAP-HA signal was measured at the kDNA and next to the kDNA, respectively. **(D)** Blue Native PAGE analysis probing for HA-tagged mtRNAP in noninduced control cells (−Tet) and mtTF1 RNAi-induced cells (+Tet [2d]). **(E)** western blot analysis of mtRNAP-HA levels in noninduced control cells (−Tet) and mtTF1 RNAi-induced cells (+Tet [2d]). The western blot was probed against HA to detect mtRNAP and against mtHSP70, which serves as a loading control. Scale bars = 5 µm. Data are shown as mean +/− standard deviation (SD). *n* = 3 for A, D and E. *n* = 100 for B and C. Statistical significance was determined using an unpaired two-tailed Student *t* test. *p* < 0.05 (*), *p* < 0.01 (**), *p* < 0.001 (***). The data underlying this Figure can be found in S1 Data.

### mtRNAP and mtTF1 are involved in maxicircle replication and transcription of mini- and maxicircles

The function of mtRNAP and mtTF1 in kDNA replication and maxicircle transcription was investigated with qPCR and RT-qPCR, respectively. Similarly to what was previously published by Grams and coworkers, we observed a decrease of maxicircle abundance but not minicircle DNA abundance two days after mtRNAP RNAi induction [33]. Maxicircle DNA (quantified with primers amplifying ND4 and 12S rDNA) was reduced below 60% upon mtRNAP RNAi (Fig 6A). RNA sequencing was performed to obtain an overview of mini- and maxi-circle transcript level changes following mtRNAP and mtTF1 knockdown. The results are shown in S5 Fig. We observed differential effects on individual mitochondrial transcripts. To further explore this observation, we performed RT-qPCR in biological triplicates on never-edited transcripts and found strand-dependent differences in maxicircle transcript levels (Fig 6B). While transcripts from the major strand (12S rRNA and ND4) went down to roughly 60%–80% 2 days after induction of mtRNAP RNAi, the minor strand transcripts (COI and ND1) were more affected by mtRNAP knockdown with less than 30% detected in induced cells compared to control cells (Fig 6B). With northern blot probing for the ND7 gRNA, we could show that mtRNAP is involved in gRNA synthesis which is in line with results from Hashimi and coworkers [34]. This probe was chosen based on its high abundance in RNA sequencing experiments of control cells compared to mtRNAP depleted cells (+Tet [2d]).

**Fig 6:**
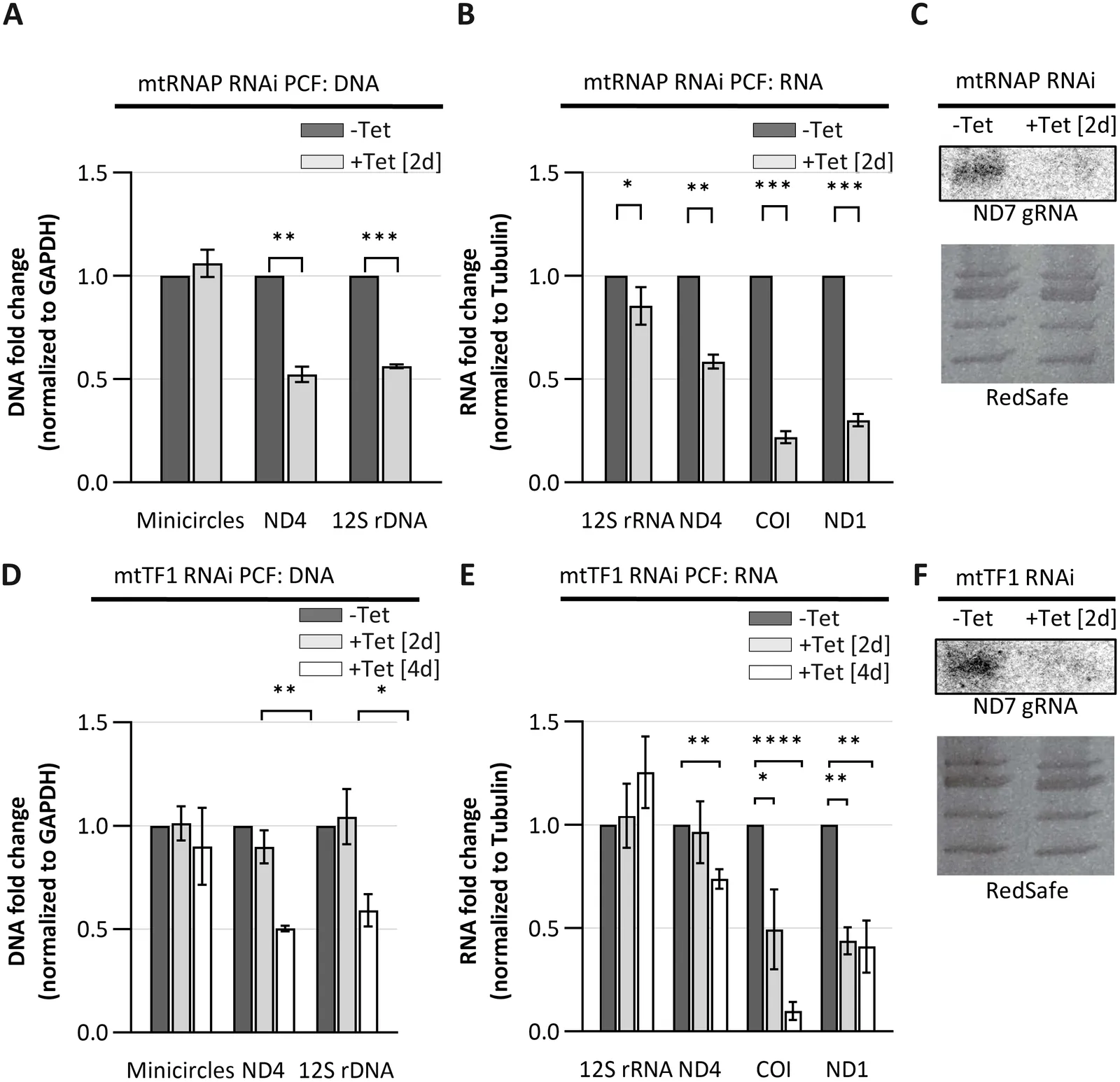
mtRNAP and mtTF1 are involved in maxicircle replication as well as maxi- and minicircle transcription in the PCF. **(A** and **B)** qPCR and RT-qPCR analysis of mini- and maxicircle DNA in control (-Tet) and mtRNAP RNAi-induced cells (+Tet [2d]). **(C)** northern blot analysis probing for ND7 gRNA in control (−Tet) and mtRNAP RNAi-induced cells (+Tet [2d]). RedSafe-stained gels serve as loading control. **(D** and **E)** qPCR and RT-qPCR analysis in control (−Tet, dark gray) and mtTF1 RNAi-induced cells (+Tet [2d] in light gray and +Tet [4d] in white). **(F)** northern blot probing for ND7 gRNA in control (−Tet) and mtTF1 RNAi-induced cells (+Tet [2d]). RedSafe-stained RNA serves as loading control. *n* = 3 for graphs A, B, E and F. Data are shown as mean +/− standard deviation (SD). Statistical significance was determined using an unpaired two-tailed Student *t* test. *p* < 0.05 (*), *p* < 0.01 (**), *p* < 0.001 (***). The data underlying this Figure can be found in S1 Data.

To exclude the possibility that the effects on the major strand upon mtRNAP RNAi were simply because of the decreased maxicircle DNA abundance, we compared the mtRNAP RNAi data with knockdown of the other primase that has been shown to result in decreased maxicircle DNA abundance, which is Primase 1 (Pri1, Tb927.8.2550). Pri1 was described by Hines and Ray in 2010 [40]. In contrast to mtRNAP, which localizes to the kDNA, Pri1 shows localization at the antipodal site (APS) throughout the cell cycle in the PCF (S6A Fig). RNAi of Pri1 results in efficient depletion of the protein as determined by Western Blot and a growth phenotype starting 3–4 days after RNAi induction (S6B Fig). Interestingly, upon Pri1 RNAi, we observed fragmentation of the kDNA 2 days after RNAi induction (S6C Fig, arrows) and subsequent shrinkage of the kDNA (S6C and S6D Fig). Similarly to the results of Hines and Ray, qPCR confirmed a loss of maxicircle DNA below 50% 2 days after Pri1 RNAi induction. In contrast to the mtRNAP RNAi, Pri1 RNAi also affects minicircles that were reduced below 60% (S6E Fig). Importantly, RT-qPCR did not show any effects on major or minor strand maxicircle transcripts. Therefore, we conclude that (i) the reduction of maxicircle DNA upon mtRNAP RNAi does not directly result in decrease of the maxicircle transcripts (ii) mtRNAP can compensate for the 50% loss of maxicircle DNA upon Pri1 RNAi, still synthesizing normal transcript levels of maxicircles.

Next, we performed qPCR and RT-qPCR to determine if mtTF1 is involved in replication and/or transcription of the kDNA. Two days after mtTF1 RNAi induction, we did not observe changes in mtDNA abundance (Fig 6D). However, 4 days after RNAi induction, a time point just at the onset of the growth phenotype, the maxicircle DNA abundance decreased to 50%. Furthermore, 2 days after mtTF1 RNAi induction, we observed a decrease of transcripts encoded on the minor strand (COI and NDI), while major strand transcripts (12S rRNA and ND4) were not affected (Fig 6E). Interestingly, after two more days of mtTF1 knockdown, the major strand seemed to be affected as well with ND4 transcript levels decreasing roughly to 60%. The effects observed for mtTF1 RNAi are very similar to the knockdown of mtRNAP with an effect on maxicircle replication and transcription with strand-specific differences. Major strand transcription seems to be affected less by RNAi when compared to the minor strand. Northern blot with probes against ND7 gRNA further showed that mtTF1 is involved in minicircle transcription, as ND7 gRNA levels were reduced upon mtTF1 RNAi (+Tet [2d]) (Fig 6F).

### A C-terminally tagged version of mtTF1 is functional and binds to mini and maxicircle DNA

In order to test if the c-terminal tag of mtTF1 interferes with its function we expressed a recoded version of the protein with a C-terminal strep- or HA tag in the mtTF1-RNAi background. The recoded protein localized to the mitochondrial fraction in biochemical fractionations as well as in immunofluorescence microscopy (S7A and S7B Fig). In these cell lines depletion of the endogenous mtTF1 did not lead to a growth defect suggesting the C-terminal tag does not interfere with its function (S7C and S7D Fig). We then used a C-terminally tagged version of mtTF1 in mtDNA immunoprecipitation experiments. After 2 days of expression of mtTF1 the protein localized preferentially at the kDNA (Fig 7A). After formaldehyde crosslinking, we isolated the co-purified DNA and quantified the presence of mitochondrial DNA by qPCR (Fig 7B), essentially as described previously [41]. We could show that mtTF1-Myc did preferentially bind to mini and maxicircle DNA when compared to a control (Fig 7B). In conclusion, functional complementation and mtDNA immunoprecipitation analyses demonstrate that C-terminally tagged mtTF1 is functional, localizes to mitochondria and the kDNA, and specifically associates with both mini- and maxi-circle mitochondrial DNA.

**Fig 7:**
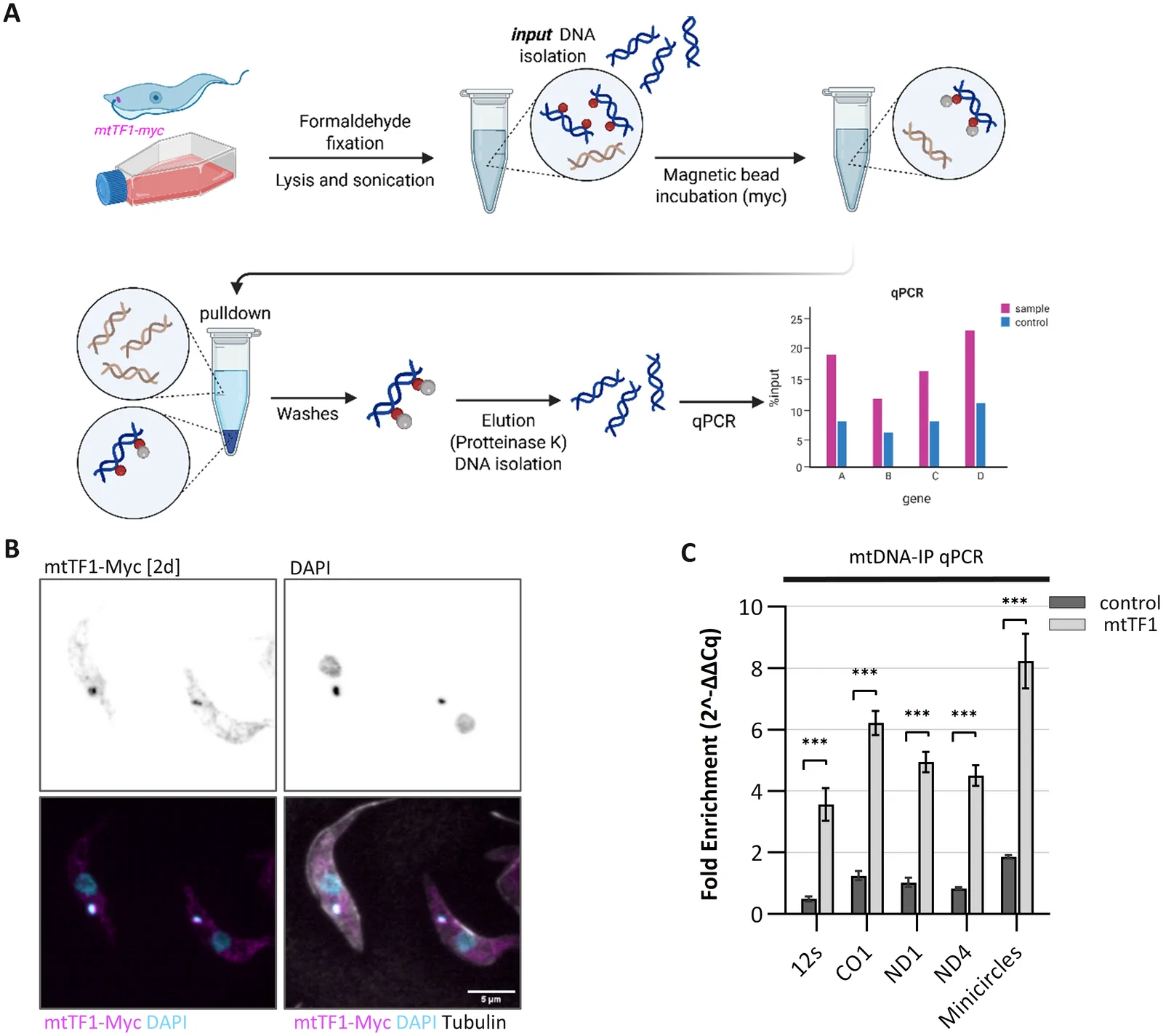
mtTF1 enriches for kDNA specific genes in a mtDNA immunoprecipitation assay. **(A)** Cells expressing a Myc tagged mtTF1 where fixed in formaldehyde, lysed and sonicated. DNA is isolated from a fraction of the lysate and analyzed by qPCR (input). Proteins are incubated and pulled down with magnetic Myc beads. Washes ensure retention of only beads bound protein-DNA complexes. DNA is eluted via protein digestion. qPCR is performed on the resulting pulled down DNA (CHIP fraction). Results are presented as a percentage of input per condition. Values are normalized to nonspecific DNA sequences. **(B)** Immunofluorescence microscopy images showing Myc-tagged mtTF1 (magenta); DNA is stained with DAPI (cyan); tubulin in gray. **(C)** qPCR analysis of mtDNA immunoprecipitation from wt cells (dark gray) and cells expressing mtTF1-myc (+Tet [2d], light gray). Data normalized to housekeeping gene 18s. Statistical significance was determined using an unpaired two-tailed Student *t* test in R. *p* < 0.05 (*), *p* < 0.01 (**), *p* < 0.001 (***). The data underlying this Figure can be found in S1 Data. Created in BioRender. Santini, E. (2026) https://biorender.com/wo3324e.

## Discussion

This study describes the identification of a previously uncharacterized protein that interacts with the mitochondrial RNA polymerase (mtRNAP, Tb927.11.5780) and is designated here as mitochondrial transcription factor 1 (mtTF1, Tb927.6.4510) in the unicellular eukaryotic parasite *T. brucei*. The protein was identified in immunoprecipitation experiments using the mitochondrial RNA polymerase (mtRNAP) as bait in both bloodstream and insect stage *T. brucei*. In both datasets, only a small number of proteins were significantly enriched, with mtTF1 and mtRNAP representing the only consistently and significantly enriched factors. Given the pronounced differences in mitochondrial biology between the two life cycle stages, it is plausible that stage-specific regulatory factors contribute to the composition or regulation of the mtRNAP complex. Future studies will determine whether any of the additional candidate proteins are bona fide components of the mtRNAP complex or represent nonspecific contaminants. While evidence for the mitochondrial localization of Tb927.6.4510 was previously shown in two independent high-throughput screens and has been further corroborated in a recent bioRxiv study [35,42,43] we now for the first time demonstrate that mtTF1 physically and functionally interacts with the mtRNAP forming a high molecular weight complex in situ and in vitro, and that both proteins are essential for mitochondrial gene expression and the maintenance of one specific component of the mitochondrial genome, the maxicircle DNA.

Functional interrogation via RNA interference revealed a strand-specific effect on steady state level transcript abundance following mtRNAP and mtTF1 depletion. Transcripts derived from genes located on the minor strand like cytochrome oxidase I exhibited a more pronounced reduction than those on the major strand. Interestingly, we also detect a very rapid effect on the protein level of the nuclear encoded cytochrome oxidase IV subunit which starts to decrease 1 day after depletion of the mtTF1, and thus several days before a fitness defect can be measured. (see Fig 4A). One plausible explanation for the strand specific effect would be differential promoter strength, wherein transcription from the major strand promoter is maintained more robustly under limiting conditions during RNAi knockdown. A comparable phenomenon has been reported in mammalian systems; in mouse cardiac mitochondria, reduced mtRNAP levels favor transcription initiation at the light strand promoter over the heavy strand promoter [20]. This has significant implications since the light strand promotor-initiated transcripts can serve as primers for mitochondrial DNA replication, suggesting a prioritization of the transcription machinery for replication purposes under constrained polymerase availability [5,9,20]. Whether a similar mechanism operates in *T. brucei* remains unknown. Although we could show specific binding of mtTF1 to mitochondrial DNA in mitochondrial DNA immunoprecipitation experiments (see Fig 7), a detailed mapping of promoter elements and assessment of their affinities for mtTF1 remain to be investigated. We would like to emphasize that at this point we do not know how exactly mtTF1 engages in the transcription or replication process.

It is currently still debated whether mitochondrial gene expression is mono- or poly-cistronic in *T. brucei*. Evidence for monocistronic transcription is based on the occupancy of mtRNAP with individual genes, instead of uniform binding of mtRNAP to the whole mitochondrial genome [41]. However, earlier studies using Northern blots identified polycistronic transcripts [44–46]. The RNA sequencing and RT-qPCR analyses presented above may be interpreted as being consistent with polycistronic (or multicistronic) transcription units. Specifically, transcripts encoded on the same strand respond similarly to mtRNAP and mtTF1 depletion: 12S rRNA and ND4 RNA (both located on the major strand) are less affected by mtRNAP and mtTF1 RNAi than CO1 and ND1 RNA (both encoded on the minor strand). This coordinated pattern of transcript abundance could suggest that genes on the same strand are transcribed as part of a single RNA molecule from a shared upstream promoter, consistent with polycistronic transcription. However, because our analyses measure steady-state RNA levels, these observations cannot be interpreted as direct evidence for such transcription units, and post-transcriptional mechanisms may also contribute to the observed differences.

We observed a decrease of maxicircle DNA abundance upon mtRNAP and mtTF1 RNAi. Similarly, disruption of mitochondrial transcription factors in other systems has been linked to defects in mtDNA replication. For instance, yeast Mtf1 and human TFAM and TFB2M are essential for mtDNA maintenance and knockout results in decreased mtDNA levels [47–49]. Furthermore, human TEFM is important for de novo mtDNA synthesis and mTERF may modulate mtDNA replication pausing [50,51]. Therefore, it is not surprising, that mtTF1 knockdown results in decreased maxicircle DNA levels.

In *T. brucei*, the mitochondrial primase 1 (Pri1, Tb927.8.2550) has previously been implicated in maxicircle DNA replication, while a second mitochondrial primase, Pri2 (Tb927.1.4010), was proposed to function primarily in the replication of minicircles [40,52]. However, our findings that the mtTF1–mtRNAP complex is also essential for maxicircle maintenance prompt a reassessment of these earlier assignments. Maxicircle replication is generally believed to occur within the central region of the kinetoplast DNA (kDNA) disk, whereas minicircle replication is localized to peripheral regions termed antipodal sites [53,54]. Notably, mtTF1 and mtRNAP are localized within the kDNA disk, in spatial concordance with maxicircle replication, whereas Pri1 localizes to the antipodal sites, in association with other proteins implicated in minicircle replication [53,54].

Functional studies further support this spatial-functional distinction. RNAi-mediated knockdown of Pri1 produces a phenotype characterized by extensive kDNA fragmentation, consistent with a primary defect in minicircle replication. A comparable phenotype is observed following depletion of the minicircle-specific DNA polymerase PolIC [55]. In both cases, the concurrent loss of minicircles and maxicircles, along with structural disintegration of the kDNA network, suggests that maxicircle depletion is likely a secondary consequence of compromised minicircle replication and kDNA network integrity. In contrast, knockdown of mtRNAP or mtTF1 leads to a selective reduction in maxicircle DNA abundance without affecting minicircle levels, indicating a specific and direct role in maxicircle maintenance. These findings strongly support the functional segregation of mitochondrial primases and transcription machinery with respect to distinct components of the kinetoplast genome.

Our findings establish mtTF1 as a key partner of mtRNAP. Together, the two proteins are crucial for mitochondrial transcription and replication in *T. brucei.* While mtRNAP is conserved among different eukaryotic organisms, mtTF1 appears unique to the Kinetoplastea. Given its essential role in cell survival, mtTF1 is a promising candidate for drug development in this group of human and animal pathogens.

## Materials and methods

### Transgenic cell lines

Transgenic *T. brucei* cell lines were generated using the PCF strain 29-13 and the BSF strain Nysm [56,57]. The PCF were grown in exponential phase (up to 1 × 10^7^ cells/ml) in semi-defined medium 79 (SDM-79, Bioconcept, #9-04V01-M) supplemented with 10% fetal calf serum (FCS) at 27 °C. BSF were grown in exponential phase (up to 1 × 10^6^ cells/ml) in Hirumi’s modified Iscove’s medium 9 (HMI-9) at 37 °C (with 5% CO_2_ and water) [58]. HMI-9 was supplemented with 10% FCS. For the PCF, antibiotics were added to a final concentration of neomycin (15 µg/ml), hygromycin (25 µg/ml), puromycin (1 µg/ml), and blasticidin (10 µg/ml). For BSF, antibiotics were added to a final concentration of neomycin (2.5 µg/ml), phleomycin (2.5 µg/ml), hygromycin (2.5 µg/ml), puromycin (0.5 µg/ml), and blasticidin (5 µg/ml).

Tagging was performed as described by Oberholzer and coworkers [59]. Briefly, the forward primer was designed to contain the last 100–120 bases of the 3′ end of the target gene, and the reverse primer contains the sequence of the 3′ untranslated region (UTR) after the target gene in reverse complement. Primers are listed in Table 3. Sequences were added to the primers to allow for amplification of a tag (Myc or HA) and a resistance gene (puromycin or blasticidin resistance gene) from the pMO plasmids by PCR. The amplified tag with the resistance gene was cleaned up according to the QIAquick PCR Purification Kit (QIAGEN; #28106). This PCR product was used for transfection. For the RNA interference (RNAi), a stem-loop construct containing the RNAi sequences in opposite direction was cloned into a modified pLew100 vector (pFC4) which contains a blasticidin resistance cassette [60]. The construct is inducible with tetracycline, which results in the expression of an RNA hairpin structure that is complementary to the target RNA and results in its degradation via RNAi. The RNAi targets the open reading frame of mtRNAP (position: 575–1045), mtTF1 (position: 98–567), and Primase 1 (position: 40–452). The RNAi target was selected using the RNAi selection tool from Redmond and coworkers [61]. Importantly, restriction sites were added to the primers: the forward primer contains HindIII (BioConcept, #R3104L) and BamHI (BioConcept, #R0136L), whereas the reverse primer contains XhoI (BioConcept, #R0146M) and XbaI (BioConcept, #R0145T) restriction sites. Prior to transfection, the plasmid was linearized with Not1 (BioConcept, #R3189M), and cleanup was performed using the QIAquick PCR Purification Kit (QIAGEN; #28106). Inducible, overexpressed recoded version of mtTF1-strep and mtRNAP-Myc were co-expressed with the inducible RNAi construct targeting the respective endogenous protein. Recoded proteins do not differ in AAs sequences compared to endogenous versions, yet the DNA sequence is incompatible to the RNAi harpin mediating the knock-down, rendering them RNAi-resistant.

**Table 3:** Primer list for PCR. The table includes information about the purpose for which the primers were used, the sequence of the forward and reverse primer as well as the plasmid used for amplification or cloning, respectively.

Purpose	Sequence fw primer	Sequence rv primer	Plasmid used for amplification/ cloning
mtRNAP pMO tagging	CTTCATGGACCTCCGTCGCCACGGTAAGGTCCTTCCGGATCCTCCGAAACGCGGGGATTTGGATCTTAACGTTGTCCTAAACTCCCCATATTTTTTCTCTGGTaccGGGcccCCCctcGAG	AAAGGGTCACATCTGAAACACACGAATAAAAGGGAAGCATGATGAGATTTGGAAGCACGTATGGTAGATGCTCACTGCATGTGCTATCATTCAGCCGTCATGGCGGCCGCTCTAGAACTAGTGGAT	pMO2/4M (3xMyc) and pMO2/4H (3xHA): puromycin or blasticidin resistance
mtRNAP RNAi	ACGAAAGCTTGGATCCACGAGTGCTAGGGGATGTCT	ACGATCTAGACTCGAGATAAGGCGCTGTAGTACCGC	pFC4: balsticidin resistance
mtTF1 pMO tagging	GGGGAAGATTCATGGGACGGAGAAAGTTTGCAAGCGATTGGAGGAAAATCGCATACCACTCCACCGTGTTACAGTCGAAAGGTTGGAAGCACTCAAGTTGGGTaccGGGcccCCCctcGAG	GAAAATTTGGAGATACTAACAAAAAGGAACCCCCAGCTGTACAACCTCCCCAAGGGAGTTGGGGGAGGGGGAAAATATGGCGGGACCCCACCATACGCGGTGGCGGCCGCTCTAGAACTAGTGGAT	pMO2/4H (3xHA): puromycin or blasticidin resistance
mtTF1 RNAi	ACGAAAGCTTGGATCCTGGCGATTGGATGAAGGCTT	ACGATCTAGACTCGAGCATCGCTTCGGGTGATACGA	pFC4: balsticidin resistance
mtRNAP overexpression	ACGATCTAGAATGCGGCGGTTGAGTCTG	ACGACTCGAGAGAGAAAAAATATGGGGAGTTTAGGAC	pCW69 (Ty-TEV-3xHA): ruromycin resistance
MtRNAP Recoded Ovrexpression Myc	ACGAACCGGTATGCGGCGGTTGAGTCTG	ACGAACCGGTAGAGAAAAAATATGGGGAGTTTAGGAC	pJM73 (C-terminal TEV 3 x Myc, puromycin)
mtTF1 overexpression	TATAAGCTTATGAACACATCAATTCGTCTG	TATATCTCGAGCAACTTGAGTGCTTCCAACC	pSA4 (3xHA): puromycin resistance
MtTF1 Recoded Ovrexpression strep	ACGATCTAGAATGAACACATCAATTCGTCT	ACGACTCGAGCTATTTCTCAAACTGAGGGTGAGACCA TGCACTTCCACCTGATCCACCGCCAGAACCTCCACCT TTTTCGAACTGTGGATGGCTCCAAGCAGAACCGGTCA ACTTGAGTGCTTCCAACC	pCW69 (C-terminal Twin-Strep tag inserted with primers during mtRNAP amplification, puromycin)
Primase 1 pMO tagging	TGGCCATCATACCAACGCATTGGAAGGACGGCGATGTTGAATGAAGCGGCAGACGCGGCAAACCGCGGTGGTGCCAACGGCATCCCTCCACAAGGAGCGGGTACCGGGCCCCCCCTCGAG	TCCTTTCTTTTTCTTTTTCTTTTTTTTTCAATTTTTCTTTTCTCCATCCATTCATGTCCCCCTCCTTCCCTGTGCTGCCACAAACTTATGTCGCTACGATGGCGGCCGCTCTAGAACTAGTGGAT	pMO2/4M (3xMyc), puromycin or blasticidin resistance
Primase 1 RNAi	ACGAAAGCTTGGATCCTGGCGAGGCTAGTTGCAAAGTTGGCC	ACGATCTAGACTCGAGGGCGGTATTTTGACGTACGC	pFC4: balsticidin resistance

Transfection was performed with 1 × 10^8^ PCF and 5 × 10^7^ BSF trypanosomes, respectively. Cells were harvested (2,800 rpm, 5 min, RT) and resuspended in 110 µl transfection buffer containing 10 µg of DNA [62]. Cells were transferred to a transfection cuvette and transfected with the Amaxa Nucleofector II program X-014 for PCF and X-001 for BSF trypanosomes, respectively. Upon transfection, cells were transferred to 10 ml SDM-79 (PCF) and 40 ml of warm HMI-9 (BSF), respectively. To obtain clonal cultures, these pools were diluted 1:10, 1:100, and 1:1000 and distributed in 24-well plates for each of the dilutions. Antibiotics were added 20 h (PCF) and 6 h (BSF) after transfection. Usually, clones were recovered 14 days after transfection for PCF and five days after transfection for BSF. The following PCF cell lines were generated: mtRNAP-3Myc (puromycin) x mtRNAP RNAi (blasticidin), mtTF1–3HA (puromycin) x mtTF1 RNAi (blasticidin), mtRNAP-3HA (puromycin) x mtTF1 RNAi (blasticidin), mtTF1–3HA (puromycin) x mtRNAP RNAi (blasticidin), mtTF1-Myc (blasticidin) x mtRNAP-HA (puromycin). Primase1–3Myc (puromycin) x Primase1 RNAi (blasticidin), mtRNAP-3HA overexpression (puromycin), mtTF1–3HA overexpression (puromycin), Recoded mtRNAP-Myc overexpression (puromycin) x mtRNAP RNAi and Recoded mtTF1-Strep x mtTF1 RNAi. The following BSF cell lines were generated: mtRNAP-3Myc (puromycin) x mtRNAP RNAi (blasticidin) in NYsm.

### Maximum-likelihood based phylogeny

We downloaded the protein sequences from TriTryp DB (https://tritrypdb.org/tritrypdb) and used the multiple sequence alignment tool MUSCLE to align the protein sequences for phylogenetic reconstruction [63]. For reconstruction, we used a maximum-likelihood-based phylogeny approach in combination with a Shimodaira–Hasegawa-like branch test [64]. The visualization of the tree was done using TreeDyn [65].

### Western blot

Whole-cell lysates were prepared for western blot as follows: cells were harvested at 4,000 rpm for 8 min and washed once with 1 ml 1x phosphate-buffer saline (PBS). Afterwards, cells were resuspended in 1x Laemmli buffer containing 12 mM Tris-HCl pH 6.8, 0.4% SDS, 2% glycerol, 1% β-mercaptoethanol, and 0.002% bromophenol blue in PBS. Denaturation was carried out at 95 °C for 5 min. 5 × 10^6^ cells were loaded per lane onto a 10% SDS-PAGE. The SDS-PAGE was run at 80 V until the samples reached the resolving gel. The voltage was increased to 100 V, and the SDS-PAGE run until the samples reached the end of the gel. Subsequently, proteins were transferred to a nitrocellulose membrane with semi-dry transfer. Two Whatman filter papers were soaked in transfer buffer (250 mM Tris, 192 mM glycine, 20% ethanol, and 0.01% SDS) and used below and on top of the SDS-PAGE and nitrocellulose membrane (Amersham Protran Premium Western-Blotting-Membrane, Nitrocellulose. Cytiva; #10600003). Semi-dry transfer was carried out at 10V with 120 mA (240 mA for two gels) for 1 h at room temperature. Prior to antibody incubation, unspecific binding of antibodies was reduced by blocking the membrane in 5% skim milk powder diluted in PBS containing 0.1% Tween 20 (Sigma-Aldrich; #93773, PBS-T) at room temperature for 30 min. Primary and HRP-conjugated secondary antibodies were diluted in PBS-T containing 5% milk with a dilution of 1:1000 and 1:10000, respectively. Antibodies are given in Table 2. Antibodies membrane was incubated with antibodies for 1 h at RT for each antibody with washing in between primary and secondary antibody incubation. Washing was repeated three times with PBS-T at room temperature for 5 min per each wash. After a final wash in PBS, the proteins were visualized using the SuperSignal West Femto Maximum Sensitivity Substrate (ThermoFisher Scientific; #34096). Chemiluminescent signal was detected with the Amersham Imager 600 (GE Healthcare) in semi-automated mode.

### Immunofluorescence assay

For immunofluorescence (IFA), 1 × 10^6^ *T. brucei* cells were harvested (2800 rpm, 5 min, RT). After washing once with PBS, cells were resuspended in 10 µl PBS and spread on a microscopy slide. Cells were then fixed with 4% paraformaldehyde (PFA) in PBS for 4 min at room temperature, followed by two PBS washes. Cells were permeabilized with 0.2% Triton X-100 (Sigma-Aldrich, 9036-19-5) for 4 min at room temperature, followed by two additional PBS washes. To reduce nonspecific antibody binding, cells were blocked with 4% bovine serum albumin in PBS (BSA, Sigma-Aldrich, #A2153) for 30 min at RT. Afterwards, cells were incubated with primary antibodies (Table 4) diluted in 4% BSA for 1 h at room temperature. The cells were then washed three times for 5 min with PBS and subsequently incubated with secondary antibodies (Table 4) at room temperature for 1 h followed by three more washes with PBS. The samples were mounted with 10 μl ProLong Gold Antifade Mountant with DNA stain DAPI (ThermoFisher, #P36931). Images were acquired with a widefield Leica DM5500 B microscope with a 100x oil immersion phase contrast objective. LAS AF software (Leica) and ImageJ were used for image analysis. Quantitative analysis of the fluorescent signal of mtRNAP and mtTF1 upon individual RNAi of the other protein was performed using ImageJ. Signal at the kDNA was determined by drawing a rectangle of 20 x 20 pixel size including the whole kDNA and measuring the integrated fluorescence intensity of mtRNAP or mtTF1 within this area. The rectangular shape was subsequently moved adjacent to the kDNA to measure the protein fluorescence next to the kDNA and thereby to determine if the protein mislocalizes upon depletion of its interaction partner. ImageJ was further used to determine the effect of Pri1 RNAi on the kDNA size. Here, a line was drawn to measure the diameter (largest length) of the kDNA disk as determined with DAPI staining.

**Table 4:** Antibodies used for western blot and immunofluorescence assay. The antibody dilution factor and provider are indicated.

Target	Dilution	Provider and catalogue number
ATOM40 rabbit	1:1000	Kind gift from André Schneider [66]
EF1α mouse	1:1000	Santa Cruz, #SC-21758
WhileIV	1:1000	Kind gift from André Schneider [67]
mtHSP70	1:1000	Kind gift from Luise Krauth-Siegel
HA rat	1:1000	Roche, #11867423001
Myc mouse	1:1000	Sigma Aldrich, #M4439
Anti-mouse HRP	1:10000	Dako, #P0260
Anti-rat HRP	1:10000	Dako, #P0450
Anti-rabbit HRP	1:10000	Dako, #P2017
Anti-mouse 488	1:1000	ThermoFisher, #A11001
Anti-rat 594	1:1000	ThermoFisher, #A11007

### Digitonin extraction

Mitochondria were enriched using digitonin extraction as described by Schneider and coworkers [68]. Briefly, 5 x 10^7^ cells were harvested by centrifugation (2,000 rpm for 7 min at RT) and washed once with 1 ml PBS. The pellet was resuspended in 250 µl 1x SoTE buffer (0.6 M sorbitol, 20 mM Tris-HCl pH 7.5, 2 mM EDTA with pH 8) and transferred to a 1.5 ml tube. To selectively permeabilize the plasma membrane, 250 µl 1x SoTE containing 0.05% digitonin (BIOSYNTH; #D3203) was gently added to obtain a final digitonin concentration of 0.025%. Cells were incubated on ice for 5 min. After centrifugation at 8000 rpm, 4 °C, for 5 min, 400 µl of the supernatant (cytosolic fraction) was transferred to a new tube, and 100 µl 5x Laemmli buffer was added. The pellet, containing the mitochondria-enriched fraction, was resuspended in 400 µl 1x SoTE, followed by the addition of 100 µl Laemmli buffer. All samples were boiled at 95 °C for 5 min. For western blot analysis, 1.5 x 10^6^ cells were loaded per lane for the whole-cell fraction, cytosolic supernatant, and mitochondria-enriched pellet.

### Co-immunoprecipitation (Co-IP)

Mitochondria were isolated using digitonin extraction, as described above. Mitochondria were lysed in 200 µl lysis buffer (20 mM Tris HCl pH 7.4, 100 mM NaCl, 0.1 mM EDTA, 25 mM KCl) supplemented with 1.5% digitonin and EDTA-free Complete ULTRA Tablets EASYpack Protease Inhibitor Cocktail (Sigma Aldrich, # 5892791001) at five times the recommended concentration. Samples were incubated on ice for 15 min. For reciprocal IP experiments, this incubation was prolonged to 30 min. During this incubation, DNase and RNase treatment was performed by addition of 1 µl RNase R (NEB, # M0100S), 2.5 µl DNase I (NEB, # M0303S) and 22 µl DNase I buffer (NEB, #B0303S) according to the manufacturer protocol. Afterwards, samples were centrifuged at 14,000 rpm, 4 °C, for 15 min. The supernatant, containing the soluble mitochondrial proteins, was used as input for the immunoprecipitation. The supernatant was incubated with 25 µl prewashed Anti-HA Magnetic Beads (Sigma Aldrich, #SAE0197) for PCF and EZview Red Anti-c-Myc Affinity Gel beads (Sigma Aldrich, #E6654) for BSF. Additionally, EZview Red Anti-HA Affinity Gel beads (Sigma Aldrich, #E6779) were used for the reciprocal IP experiments. Binding was performed at 4 °C for 1 h. Beads were pelleted using either a magnetic holder or centrifugation at maximum speed, for 10 s. The supernatant (flowthrough) containing unbound proteins, was transferred to a new tube. Beads were subsequently washed five times with 500 µl wash buffer (20 mM Tris HCl pH 7.4, 100 mM NaCl, 0.1 mM EDTA, 25 mM KCl) containing 0.1% digitonin and EDTA-free complete ULTRA Tablets EASYpack Protease Inhibitor Cocktail (Sigma Aldrich, # 5892791001) at five times the recommended concentration. For subsequent mass spectrometry, three additional washes were performed using digitonin-free wash buffer. Data and further detail of the mass spectrometry protocol are publicly available on PRIDE with the dataset identifier PXD066647. For western blot analysis, samples were prepared and loaded as described above.

### RNA isolation

A total of 1 x 10^8^ cells were harvested by centrifugation at 2500 rpm for 8 min at room temperature. The pellet was washed with 1 ml PBS, transferred to a 1.5 ml tube and centrifuged again at 2500 rpm for 8 min at room temperature. After removing the supernatant, the cell pellet was resuspended in 1 ml TRIzol (Tri-Reagent, Merck, #9424). Subsequently, 200 µl of chloroform was added and the samples were vortexed vigorously. The mixture was then centrifuged at 12,000 rpm for 15 min at 4 °C. The upper aqueous phase (400 µl) was carefully transferred to a new tube containing 500 µl of ice-cold isopropanol, ensuring that the interphase was not disturbed. The tube was inverted several times to mix the solution. RNA was precipitated by centrifugation at 12,000 rpm for 15 min at 4 °C. Subsequently, the supernatant was removed and discarded. The RNA pellet was washed twice with 1 ml 70% ethanol followed by centrifugation at 7,500 rpm for 5 min at 4 °C. The ethanol was removed, and the tube was inverted and left open at room temperature to air-dry for at least 5 min. Finally, the RNA pellet was resuspended in 50 µl RNase-free water and incubated at 55 °C for 5 min on a heating block. RNA concentration was determined using a Nanodrop spectrophotometer and RNA integrity was assessed by running 1 µg of RNA on a 1% agarose gel for 40 min at 100 V. RNA was stored at −80 °C or used directly for DNase treatment and cDNA synthesis.

### Northern blot analysis for small mitochondrial RNAs

#### Isolation of mitochondrial RNA.

Cells (1 x 10^8^) were harvested by centrifugation at 2,000 rpm for 7 min at room temperature (RT) and resuspended in 1 mL PBS. Following a second centrifugation (2,000 rpm, 7 min, RT), the pellet was resuspended in 0.5 mL 1× SoTE buffer (RT). To permeabilize the plasma membrane, 0.5 mL of 0.03% digitonin in 1× SoTE was added gently, followed by incubation on ice for 10 min. Mitochondria-enriched pellets were obtained by centrifugation at 8,000 rpm for 5 min at 4 °C, with supernatants carefully removed. RNA was isolated using Tri-reagent (Sigma-aldrich, T9424), following manufacturer instructions. Briefly, 1 mL Tri-reagent was added to the pellet and vortexed until completely dissolved. After a 10 min RT incubation, 200 µL chloroform was added, vortexed for 15 s, and incubated for 3 min. Samples were centrifuged at 12,000 *g* for 15 min at 4 °C, and the upper aqueous phase (~450 µL) was carefully transferred to a new tube. RNA was precipitated with 500 µL isopropanol, incubated for 10 min at RT, and centrifuged at 12,000 *g* for 15 min at 4 °C. The RNA pellet was washed with 1 mL 75% ethanol, centrifuged at 7,500 *g* for 5 min at 4 °C, air-dried (~15 min), and resuspended in 20–50 µL RNase-free water. RNA integrity and concentration were assessed using a Nanodrop spectrophotometer.

#### Polyacrylamide gel electrophoresis and northern blot transfer.

Denaturing PAGE was performed using 15% urea-TBE gels (Bio-Rad, #4566055). Five micrograms of RNA were heated at 95 °C for 60 s and mixed with 2 × RNA loading dye (Formamide containing 0.025% (v/v) 1% bromophenol blue and 0.025% (v/v) 1% Xylene cyanole) prior to loading. Electrophoresis was conducted in 1 × TBE running buffer at a constant 200 V for 90 min. Following electrophoresis, gels were stained in 0.5 × TBE containing RedSafe for 5 min, and gel images were captured before transfer. RNA was transferred onto a charged nylon membrane via semi-dry electroblotting (300 mA, 1 h) in 0.5 × TBE buffer. The membrane was UV crosslinked at 0.12 J.

#### Probe labeling and hybridization.

The following oligonucleotides complementary to the target RNA were ordered via Microsynth:

mO236 667-716 (ND7): tattatcattcatactcactgtactataagttatctgcatcgtattatat
mO218 636 – 686 (CR3:) tatatatcatcaatctcattaccgtatataagccttcgttattttatatat

Oligonucleotide probes were 5′-end labeled using T4 polynucleotide kinase (PNK). The labeling reaction (total volume 10 µL) included 1.2 µL oligo (10 pmol/µL), 1 µL 10 × PNK buffer, 5 µL H2O, 2 µL ɣ-32P-ATP, and 0.8 µL T4 PNK (10,000 U/mL). The reaction was incubated at 37 °C for 30 min. Membranes were prehybridized in 15–20 mL hybridization buffer at 37 °C before adding the radiolabeled probe. Hybridization was carried out at 37 °C for ~20 h.

### Washing and imaging

Following hybridization, membranes were washed sequentially in prewarmed 2 × SSC (0.1% SDS) for 10 min at 37 °C, 0.5 × SSC (0.1% SDS) for 10 min at 37 °C, and briefly rinsed in water at RT. The membranes were air-dried, wrapped in plastic wrap, and exposed to a phosphor screen for ~72 h. RNA signals were visualized using phosphor imaging.

### DNA isolation

5 × 10^7^ cells were harvested by centrifugation at 2,000 rpm for 5 min. After removing the supernatant, the cells were washed in 1 ml PBS and transferred to a 1.5 ml tube. Following centrifugation at 3,000 rpm for 5 min, the pellet was resuspended in 100 µl DNA QuickExtract DNA Extraction Solution (LubioScience, #QE0905T) and vortexed for 15 s. Samples were then incubated at 65 °C for 6 min, vortexed again for 15 s, and incubated at 98 °C for 2 min. The DNA concentration was measured using a Nanodrop spectrophotometer. Here, the 260/280 ratio was typically below 1. The reproducibility of this method was verified by comparing mtRNAP and Primase 1 RNAi data with published literature [33]. In addition to being extremely fast, the method was verified to be reproducible. DNA concentration was adjusted to 100 ng/µl and stored at −20 °C. For qPCR, a DNA working concentration of 4 ng/µl was used.

### RNA-sequencing, qPCR and RT-qPCR

For DNase treatment, a total amount of 10 µg RNA was used. In a final volume of 100 µl, 10 µl DNase buffer (NEB, #B0303S), 1 µl DNase (NEB, #M0303S), and 10 µg RNA were incubated at 37 °C for 15 min. Afterwards, the RNeasy MinElute Cleanup kit (QIAGEN, #74204) was used to purify the RNA prior to cDNA synthesis. It is crucial to obtain pure RNA for reproducibility of RT-qPCR results. After cleanup, the RNA was sent to FASTERIS for RNA sequencing (one replicate for mtRNAP and mtTF1 RNAi (each -Tet and +Tet [2d]). The RNA sequencing data and further information about the RNA sequencing protocol and analysis are publicly available at NCBI GEO with the dataset identifier GSE297664.

For RT-qPCR, the iScript gRNA Clear CDNA synthesis Kit (Biorad, #1725034) was used to synthesis cDNA according to the manufacturer’s protocol. Briefly, 1 µg RNA was used and adjusted to a final volume of 40 µl with water. Subsequently, 10 µl of 5x iScript Super Mix was added, either containing reverse transcriptase or lacking it for the negative control. The cDNA synthesis was performed in a thermocycler using the following program: 25 °C for 5 min, 46 °C for 20 min, 95 °C for 1 min, and hold at 4 °C. cDNA was stored at −20 °C. The cDNA was then diluted 1:10 with water and directly used for RT-qPCR.

All primers used for qPCR and RT-qPCR are listed in Table 5. Primers for detecting minicircles were previously published by Gluenz and coworkers [54], while primers for ND4 were previously published by Li and coworkers [69]. GAPDH and actin primer were previously published by Brenndörfer and Boshart [68]. Primers for the detection of β-tubulin, 12S rRNA, COI, ND1, and Primase 1 were designed using the IDT PrimerQuest Tool (https://eu.idtdna.com/PrimerQuest/Home/Index). For each target, three cycle threshold (Cq) values were averaged (pipetting triplicates). Each qPCR analysis was performed in biological triplicates. The comparative Cq method (2^(−ΔΔCq)) was used. Briefly, raw Cq values of target genes were normalized to Cq values of the housekeeping genes (GAPDH or tubulin). Importantly, GAPDH was only used as a housekeeping gene for qPCR but not RT-qPCR, as its mRNA levels increased upon mtRNAP and mtTF1 RNAi. To ensure the validity of tubulin as a housekeeping gene, actin was always run in parallel for RT-qPCR and showed stable expression across conditions. Final Cq values were then further normalized to the uninduced control group for better visualization and are presented as fold change.

**Table 5:** Primers used for qPCR and RT-qPCR. Given are the forward (fw) and reverse (rv) primer for each target detected with qPCR or RT-qPCR.

Target	Sequence fw primer	Sequence rv primer
Minicircle	TAT GGG CGT GCA AAA ATA CA	CGA AGT ACC TCG GAC CT
12S rRNA	TTT AAG GTT GGT AGG CAT AGT T	CCC AAC CAC AAA TCC TTT ACA T
ND4	CAA TCT GAC CAT TCC ATG TGT GA	TTT CAG CAC AAT ACT TGC TAA TAA AAC A
CO1	TGG TTG GGT TTC CCG ATA TG	CTA AGC AAC CAA ATC CTC CAA TAA A
ND1	TGG ACT GCT TCT TGA TGG ATT AC	AGT AAC AAG GCC AGC AAC AA
Actin	GTA CCA CTG GCA TTG TTC TCG	CTT CAT GAG ATA TTC CGT CAG GTC
GAPDH	AGA TTG ATG TCG TTG CTG TTG TG	ATG GCT TGC TCT TCG TAG TCG
β -Tubulin	GAC TTT CTC CAT CCC ATC C	CAC ATC GAC TCAT CGG AGT TT
Primase 1	ACC AGT TCC ACA AGA GAA TAC C	GCT GGT GTG ATT CTC CTT CATA
mtTF1	ATG GGA TGTTGC TGT GGA G	CCC AAT CGA GTT CTT CGA TCA
mtRNAP	CTC TAT GAT GGC GTC GGA AAT A	GAG CGG TGC TGGT CAC TAA A
18s	CGG AAT GGC ACC ACA AGA C	TGG TAA AGT TCC CCG TGT TGA

The qPCR and RT-qPCR were performed as follows: First, primer mixes were prepared for each target. Primers (stock concentration of 100 µM) were diluted to a final concentration of 1.5 µM by adding 15 µl of each primer into 970 µl of water. Subsequently, the primer mix was combined with the Sso Advanced Universal SYBR Green Supermix (Bio-Rad, #1725271) in a 3:5 ratio. For the RT-qPCR and qPCR, 2 µl of the cDNA (diluted 1:10 after cDNA synthesis) and DNA (at a concentration of 4 ng/µl) were pipetted into a 96-well plate. Then, 8 µl of the SYBR Green Supermix containing the primer mix was added, and the plate was sealed. The thermocycler (Bio-Rad C1000 Touch PCR/Thermal Cycler) program starts at 95 °C for 5 min. DNA is amplified during 40 cycles, with each cycle consisting of denaturation at 95 °C for 10 s and annealing and elongation at 60 °C for 15 s. Fluorescent intensity was measured after each cycle. During the final step, melting curves were obtained by gradual increasing the temperature from 60 °C to 95 °C.

### Blue native PAGE

Mitochondria were isolated from 1 x 10^8^ cells as described above using digitonin extraction. The mitochondria were then lysed with digitonin solubilization buffer (20mM Tris-HCl pH 7.4, 50 mM NaCl, 5% Glycerol, 0.05mM EDTA, 1 mM PMSF) containing 1% digitonin. Lysis was carried out on ice for 15 min. Subsequently, samples were centrifuged at 14,000 rpm, 4 °C for 15 min. The supernatant was then used for the Blue Native PAGE, which was performed as previously described [70]. A total of 5 x 10^7^ cells were loaded per lane on a native gel with a gradient of 4–13%.

### Pre-gelation staining expansion microscopy (PS-ExM)

The pre-gelation staining expansion microscopy was performed according to Atchou and coworkers [39]. Briefly, *T. brucei* cells were stained as described above for regular IFA. The only difference is that 100 µl of washed cells (1 × 10^6^) was settled on poly-D Lysin functionalized 12 mm coverslips for 15 min and directly fixed with 100 µl 4% of PFA for 4 min (PFA is thereby diluted to a final concentration of 2%). After permeabilization and antibody incubation, cells were incubated in PBS containing 0.7% formaldehyde (FA, 36.5%–38%, SIGMA, #F8775) and 1% acrylamide (AA, 40%, SIGMA, #A4058) for functionalization. AA will later allow to link the cells to the hydrogel. Subsequently, samples are embedded into the hydrogel: the gelation solution was prepared freshly with 0.5% ammonium persulfate (APS, 17874, ThermoFisher) and 0.5% tetramethyl ethylenediamine (TEMED, ThermoFisher, #17919) added to the monomer solution containing 19% sodium acrylate (SA, 97%–99%, SIGMA, #408220), 10% acrylamide (AA, 40%, SIGMA, #A4058) and 0.1% N,N’-methylenebisacrylamide (BIS, 2%, SIGMA, #M1533). For gelation, 35 µl of the gelation solution was used per coverslip, and the samples were incubated on ice for 5 min, followed by incubation at 37 °C for 30 min. The gels were denatured in 1 ml denaturation buffer (200 mM sodium dodecyl sulfate SDS, 200 mM NaCl and 50 mM Tris in deionized water, pH 9). After gently shaking the gels at room temperature for 15 min, the gels detached from the coverslip. Gels were transferred to 1.5 ml tubes and denatured in 1 ml of denaturation buffer at 95 °C for 30 min. After a brief incubation of the gels in PBS, the DNA was stained with 5 µg/ml 4′,6-diamidino-2-phenylindole (DAPI, SIGMA, #D9542-5MG) diluted in 2% bovine serum albumin (BSA). DAPI staining was performed at 37 °C with gentle agitation for 1 h. Finally, the hydrogels were expanded in deionized water for 1 h. After the final expansion, the gels were cut and mounted on poly-D-lysine functionalized 35 mm glass-bottom dishes (Cellvis, #D35-20-1.5-N. 35 mm glass bottom dish with 20 mm micro-well #1.5 cover glass). Images were acquired with a 60x oil objective (NA = 1.4) on a NIKON Ti 2 CREST V3 equipped with a Hamamatsu Flash 4.0 camera and a Celesta light engine in widefield mode. Images were acquired with a z-step size of 300 nm and pixel size of 108 nm. ImageJ was used for analysis. The Pearson correlation coefficient was measured with the ImageJ plugin JaCoP by selecting only the kDNA region with the rectangular selection tool (*n* = 10). To calculate the average Pearson correlation coefficient (r), the values were transformed using Fisher’s transformation z = artanh(r), which also allowed to determine the standard error of means (SEM). The obtained averag and SEM were then back transformed with the formula r = tanh(z).

### mtDNA immunoprecipitation assay

mtTF1-Myc expression was induced with tetracycline (1 µg/ml) for 48 h. Approximately 400 M cells were harvested per condition on the day of the experiment. Cells were resuspended in 40 ml of fresh SDM79 with the addition of 4 ml crosslinking solution (100 mM NaCl, 50 mM HEPES pH 7.3, 1mM EDTA, and 2.5% formaldehyde MeOH free). Cells incubated at RT for 25 min, reaction quenched by adding glycine (final concentration 125 mM), followed by centrifugation at 3,000× *g* for 15 min at 4 °C. Cell pellets were washed with cold PBS and snap-frozen in liquid nitrogen. Pellets were resuspended in lysis buffer (50 mM Tris-HCl pH 7.5, 300 mM NaCl, 5 mM EDTA, 1% NP-40). Sonication was performed with a Branson 450 sonicator (3 times 10 s active 30 s pause cycles at 10% power) in a 50 ml tube. Lysed and sonicated cells were transferred in a 1.5 ml Eppendorf tube and diluted 5 times with additional lysis buffer without detergent (50 mM Tris-HCl pH 7.5, 300 mM NaCl, 5 mM EDTA). Debris was removed by centrifugation at 21,000× *g* for 30 min at 4 °C. The supernatant was incubated with magnetic Anti-Myc-beads overnight under rotation at 4 °C, while a fraction of it was used for chloroform-phenol extraction and ethanol precipitation for DNA isolation (input fraction). Beads were sequentially washed with buffers containing increasing stringency, including high-salt and LiCl-containing wash buffers, followed by a final wash in low-detergent buffer. Wash Buffer 1 (WB1: 20 mM Tris pH 7.5, 300 mM NaCl, 1 mM EDTA, 0.1% NP40), Wash Buffer 2 (WB2: 20 mM Tris pH 7.5, 300 mM LiCl, 1 mM EDTA, 0.5% NP40, 0.5% NaDOC), Wash Buffer 3 (WB3: 10 mM Tris pH 8, 300 mM NaCl, 1 mM EDTA). Washes were repeated twice per buffer and low-binding tubes were exchanged after each buffer change. DNA–protein complexes were eluted in SDS-containing Elution Buffer (EB: 50 mM Tris pH 7.5, 300 mM NaCl, 10 mM EDTA, 1% SDS) at 65°C for 30 min. Crosslinks were reversed by incubation at 65 °C overnight in the presence of Proteinase K. DNA was purified by phenol–chloroform extraction and ethanol precipitation. Recovered DNA was analyzed by quantitative PCR using primers specific for maxicircle-encoded genes and a control region (18s). Enrichment was calculated relative to input DNA and normalized to a nontarget locus (18s).

### Recombinant mtTF1 and mtRNAP expression

#### Cloning.

The sequence encoding mtRNAP (residues 31−1274, Δ30mtRNAP) was amplified from *trypanosoma brucei* (*t. brucei*) genomic DNA (gDNA) and cloned into the 438-C vector system (kind gift from Scott Gradia; Addgene plasmid # 55220; http://n2t.net/addgene:55220; RRID: Addgene_5522043) using ligation independent cloning (LIC). Cloning of the Δ30mtRNAP gene was performed in frame with an N-terminal hexahistidine (6xHis) tag followed by a maltose binding protein (MBP) tag and a TEV cleavage site (forward primer 5′-TACTTCCAATCCAATGCAGGAGCAATAAAATCCCCAAAGTCG-3′, reverse primer 5′- TTATCCACTTCCAATGTTATTAAGAGAAAAAATATGGGGAGTTTAGGACAACG-3′ (IDT)). The full-length mtTF1 gene was amplified from *T. brucei* gDNA and cloned into the 438-A vector (kind gift from Scott Gradia; Addgene plasmid # 55218; http://n2t.net/addgene:55218; RRID: Addgene_5521843) using LIC (forward primer 5′-TACTTCCAATCCAATCGATGAACACATCAATTCGTCTGCTTTCG-3′, reverse primer 5′-TTATCCACTTCCAATGTTATTACAACTTGAGTGCTTCCAACCTTTC-3′), resulting in a construct without affinity tags. Both vectors were used for subcloning a co-expression vector encoding the 6xHis-MBP-Δ30mtRNAP and mtTF1 proteins. This co-expression vector was used as template for amplification of Δ130mtRNAP (residues 131−1274) and mtTF1 which was cloned into 438-C vector using LIC (forward primer 5′-TACTTCCAATCCAATGCAGAGAGTGTGCTCTCTGTTATTG-3′, reverse primer 5′- TTATCCACTTCCAATGTTATTACAACTTG-3′), resulting in a co-expression vector encoding 6xHis-MBP-Δ130mtRNAP and mtTF1 proteins. The final vector sequences were confirmed by Oxford Nanopore Technology sequencing (Microsynth Seqlab).

#### Expression.

The mtRNAP-mtTF1 complex was recombinantly expressed in a baculovirus-insect cell system as previously described [71,72]. Briefly, DH10EmBacY cells were transformed with Δ130mtRNAP-mtTF1 vector by electroporation. Positive clones as identified by blue-white screening were used for V0 bacmid extraction with isopropanol precipitation. The bacmids were transfected into Sf9 cells grown in ESF921 medium (Expression Technologies) using the X-tremeGENE9 transfection reagent (Sigma) and incubated for 3–5 days at 27 °C. V1 virus was generated by infection of Sf21 cells with V0 virus and used for infection of Hi5 cells for large-scale protein expression. Sf21 and Hi5 cells were cultured in ESF921 medium (Expression Technologies) at 27 °C and 60 rpm for 3–5 days. Afterwards, the cells were pelleted by centrifugation to allow collection of the virus or harvesting of the cells. The virus was stored at 4 °C and the cells were snap frozen in liquid N2 for storage at −80 °C.

#### Purification.

Cell pellets were resuspended in lysis buffer (50 mM HEPES pH 7.5, 500 mM NaCl, 5 mM MgCl2, 10% Glycerol, 5 mM DTT) and supplemented with cOmplete, EDTA-free Protease Inhibitor Cocktail (Sigma-Aldrich). The cell suspension was lysed by sonication and centrifuged for 1 h at 47,808 rcf and 4 °C. The supernatant was incubated with Amylose resin (NEB) equilibrated in lysis buffer for 1 h at 4 °C on a rotary wheel. The resin was washed five times with lysis buffer, before the complex was eluted by addition of TEV protease (homemade) and ON incubation at 4 °C on a rotary wheel. The eluate was diluted to a final NaCl concentration of 200 mM with buffer A (50 mM HEPES pH 7.5, 10% Glycerol, 5 mM DTT) and applied to a HiTrap Q HP 5 ml column (Cytiva) equilibrated in a mixture of buffer A and 10% buffer B (50 mM HEPES pH 7.5, 2 M NaCl, 10% Glycerol, 5 mM DTT). The complex was eluted using a linear gradient from 200–1,000 mM NaCl. Peak fractions, as identified by SDS-PAGE, were pooled and concentrated using a MWCO 30,000 Amicon Ultra Centrifugal Filter (Merck). The concentrated sample was applied to a Superose 6 Increase 10/300 GL column (Cytiva) equilibrated with lysis buffer. Fractions containing the Δ130mtRNAP-mtTF1 complex were concentrated as described above and snap frozen in liquid N2 for storage at −80 °C. The identity of purified Δ130mtRNAP and mtTF1 was verified by mass spectrometry.

#### Analytical size exclusion chromatography.

32-µg of purified Δ130mtRNAP-mtTF1 complex was applied to a Superose 6 Increase 3.2/300 column (Cytiva) equilibrated in lysis buffer. Peak fractions were precipitated by 10 min incubation with 0.25 sample volumes trichloroacetic acid at 4 °C. Precipitated protein was spun down by centrifugation and washed with 200 µl cold acetone. Remaining acetone was dried off on a heat block. The protein pellet was resuspended in 4 µl buffer (20 mM HEPES pH 7.5, 200 mM NaCl, 2 mM DTT) supplemented with mPAGE LDS Sample buffer (Merck) and examined by SDS-PAGE.

## Supporting information

S1 Fig mtRNAP-Myc expression does not rescue endogenous mtRNAP depletion by RNAi. **(A)** western blot from digitonin extraction from cells expressing mtRNAP-Myc; total cell lysate (T), soluble cytosolic faction (S) mitochondrial-enriched pellet (P). EF1α and HSP70 serve as cytosolic and mitochondrial marker, respectively. **(B**) Immunofluorescence microscopy images showing mtRNAP, magenta; DNA, (cyan), tubulin, gray. Additionally, inverted black and white images are shown for better visualization. **(C)** RT-qPCR mtRNAP two days post RNAi induction (+Tet 2d) **(D)** Growth curve of control cells (−Tet, dark gray) and cells expressing mtRNAP-Myc and mtRNAP RNAi (+Tet, orange). Inset: western blot of total cell extract with mtRNAP-Myc in noninduced (−Tet) and induced cells (+Tet [2d]). Data are shown as mean +/− standard deviation (SD). *n* = 3 for C and D. Statistical significance was determined using an unpaired two-tailed Student *t* test. *p* < 0.05 (*), p < 0.01 (**), *p* < 0.001 (***).The data underlying this Figure can be found in S1 Data. Note that the blots containing mtRNAP-myc were cut and only the illustrated portions could be retrieved.
(TIF)

S2 Fig Workflow of the immunoprecipitation of the HA-tagged mtRNAP to identify interaction partners. Cells with C-terminally HA-tagged mtRNAP were lysed with a low percentage of digitonin, and mitochondria were enriched by centrifugation. Following mitochondrial lysis, the proteins (Input) were incubated with magnetic anti-HA beads for 1 h at 4 °C. The immunoprecipitation (IP) fraction contains the mtRNAP and potential interaction partners, while other mitochondrial proteins are in the flowthrough (FT). Created in BioRender. Santini, E. (2026) https://biorender.com/wo3324e.
(TIF)

S3 Fig Immunoprecipitation analysis of mtTF1-myc verifies mtRNAP interaction. **(A)** western blot analysis of the immunoprecipitation performed with PCF cells expressing myc-tagged mtTF1. Enriched mitochondrial fraction was used as input for the immunoprecipitation. The mitochondrial protein VDAC serves as a control; FT, flowthrough; IP, immunoprecipitation fraction. **(B)** Immunofluorescence assay images of the cells used for the IP, showing mtTF1 (induced 1d), magenta; DNA, (cyan), tubulin, gray. Additionally, inverted black and white images are shown for better visualization. **(C)** Volcano plot of quantitative mass spectrometry data from the mtTF1-myc IP. Horizontal lines indicate a *p*-value of 0.0005 and vertical lines mark a 16-fold enrichment; *n* = 3. The data underlying this Figure can be found in S1 Data.
(TIF)

S4 Fig Additional data for mtTF1-HA x mtRNAP RNAi and mtRNAP-HA x mtTF1 RNAi. **(A)** Growth curve of noninduced control cells (−Tet) and mtRNAP RNAi-induced cells (+Tet). Cells express a C-terminally HA-tagged mtTF1. **(B)** Blue Native PAGE analysis of the mtRNAP and mtTF1 high molecular weight complex. The C-terminally HA-tagged mtRNAP and mtTF1 were detected with antibodies. Coomassie staining below the Blue Native PAGE serve as loading control. **(C)** Growth curve of noninduced control cells (−Tet) and mtTF1 RNAi-induced cells (+Tet). Cells express a C-terminally HA-tagged mtRNAP. **(D)** Blue Native PAGE analysis of noninduced control cells (−Tet) and mtTF1 RNAi-induced cells (+Tet [2d]). The C-terminally HA-tagged mtRNAP was detected with antibodies. Coomassie staining below the Blue Native PAGE serve as loading control. *n* = 3 for B and C. Statistical significance was determined using an unpaired two-tailed Student *t* test. *p* < 0.05 (*), *p* < 0.01 (**), p < 0.001 (***). The data underlying this Figure can be found in S1 Data.
(TIF)

S5 FigData analysis of RNA sequencing upon mtRNAP and mtTF1 knockdown.**(A)** Overview of never-edited, maxicircle-encoded transcripts on the major (blue) and minor (red) strand. **(B** and **D)** Maxicircle transcript foldchange upon mtRNAP and mtTF1 RNAi (+Tet[2d]) shown relative to noninduced control cells. Maxicircle major strand transcript are shown in blue and minor strand transcripts in red. *n* = 1. **(C** and **E)** gRNA foldchange upon mtRNAP and mtTF1 RNAi. Shown is the mean +/- SEM of different gRNAs combined that had a read coverage ≥ 5. Statistical significance was determined using an unpaired two-tailed Student *t* test. p < 0.05 (*), *p* < 0.01 (**), *p* < 0.001 (***). The data underlying *t*his Figure can be found in S1 Data.(TIF)

S6 Fig Primase 1 localizes to the antipodal sites flanking the kDNA throughout the cell cycle and is essential for cell viability and kDNA replication in the PCF. **(A)** Immunofluorescence images of cells harboring a C-terminally Myc-tagged Primase 1 (Pri1, green) together with DAPI staining (DNA, blue). Cells are visualized with DIC (differential interference contrast microscopy). Images show different cell cycle stages. N = nucleus, k = kDNA. **(B)** Growth curve and western blot analysis of noninduced (−Tet) and Pri1 RNAi-induced (+Tet) cells. ATOM40 served as a loading control. **(C)** Microscopy images showing DAPI staining (DNA, cyan) of noninduced control cells (−Tet) and Pri1 RNAi-induced cells (+Tet [2 and 4d]). Cells are visualized with DIC (differential interference contrast microscopy). Arrow heads point at fragmented kDNAs **(D)** kDNA size quantification in control cells (−Tet, gray) and Pri1 RNAi-induced cells (+Tet [2d] in red and +Tet [4d] in dark red). **(E** and **F)** qPCR and RT-qPCR analysis in noninduced control cells (−Tet, dark gray) and upon Pri1 RNAi (+Tet [2d], light gray). Scale bars = 5 µm. Data are shown as mean +/− standard deviation (SD). *n* = 3 for B, E, and F. *n* = 100 for D. Statistical significance was determined using an unpaired two-tailed Student *t* test. *p* < 0.05 (*), *p* < 0.01 (**), *p* < 0.001 (***). The data underlying this Figure can be found in S1 Data.
(TIF)

S7 Fig mtTF1-strep expression rescues endogenous mtTF1 depletion by RNAi. **(A)** western blot from digitonin extraction from cells expressing mtTF1-strep; total cell lysate (T), soluble cytosolic faction (S) mitochondrial-enriched pellet (P). EF1α and HSP70 serve as cytosolic and mitochondrial marker, respectively. **(B)** Immunofluorescence microscopy images showing mtTF1, magenta; DNA, (cyan), tubulin, gray. Additionally, inverted black and white images are shown for better visualization. **(C)** RT-qPCR mtTF1 two days post RNAi induction (+Tet 2d) **(D)** Growth curve of control cells (-Tet, dark gray) and cells expressing mtTF1-Strep and mtTF1 RNAi (+Tet, orange). Inset: western blot of total cell extract with mtTF1-Strep in noninduced (-Tet) and induced cells (+Tet [2d]). Data are shown as mean +/- standard deviation (SD). *n* = 3 for C and D. Statistical significance was determined using an unpaired two-tailed Student *t* test. *p* < 0.05 (*), *p* < 0.01 (**), *p* < 0.001 (***). The data underlying this Figure can be found in S1 Data. Note that only a part of the blots containing mtTF1-Strep were retrieved.
(TIF)

S8 Fig Purification of recombinant mtRNAP-mtTF1 complex. **(A)** Analytical size exclusion chromatography profile of the recombinant mtRNAP-mtTF1 complex using a Superose 6 Increase 3.2/300 column. The absorbance at 260 and 280 nm is shown in orange and blue, respectively. **(B)** SDS-PAGE analysis of the peak fractions from (A) using a 4%–12% polyacrylamide gel. The data underlying this Figure can be found in S1 Data.
(TIF)

S1 Raw Images Collection of uncropped and minimally edited western blots and blue native pages for transparency reasons. (PDF)

S1 Table Significantly enriched proteins of mtRNAP immunoprecipitation from PCF cells. (XLSX)

S1 Data Collection of unprocessed data found in the manuscript figures. (XLSX)

S2 Table Significantly enriched proteins of mtRNAP immunoprecipitation from BSF cellscells. (XLSX)

## Abbreviations

APS: antipodal site
BSA: bovine serum albumin
BSF: bloodstream form
Co-IP: co-immunoprecipitation
DBMR: Department for BioMedical Research
FCS: fetal calf serum
IFA: immunofluorescence analysis
IP: isoelectric point
kDNA: kinetoplast DNA
LIC: ligation independent cloning
LSP: light strand promoter
MBP: maltose binding protein
MIC: Microscopy Imaging Centre
Mtf1: mitochondrial transcription factor
mtDNA: mitochondrial DNA
mTERF: mitochondrial transcription termination factor
mtRNAP: mitochondrial RNA-polymerase
OriH: heavy strand origin of replication
OriL: light-strand origin
PBS: phosphate-buffer saline
PCF: procyclic form
PMSCF: Proteomics and Mass Spectrometry Core Facility
PNK: polynucleotide kinase
PS-ExM: Pre-Staining Expansion Microscopy
PFA: paraformaldehyde
RNAi: RNA interference
RT: room temperature
SD: standard deviation
SEM: standard error of means
TEFM: transcription elongation factor
TFAM: transcription factor A
TFB2M: transcription factor B2
U-ExM: Ultrastructure Expansion Microscopy
UTR: untranslated region.
