# Supplementary material for: The kinetoplastid-specific transcription factor mtTF1 is essential for mitochondrial gene expression and maxicircle replication in Trypanosoma brucei
Source: PLoS Biol. 2026 Sep 21;24(9):e3003945. doi: 10.1371/journal.pbio.3003945 (PMC13623095; doi:10.1371/journal.pbio.3003945)

Figure 1: mtRNAP localizes to the kDNA and is essential in the procyclic form (PCF) and bloodstream form (BSF) of trypanosomes. (A)

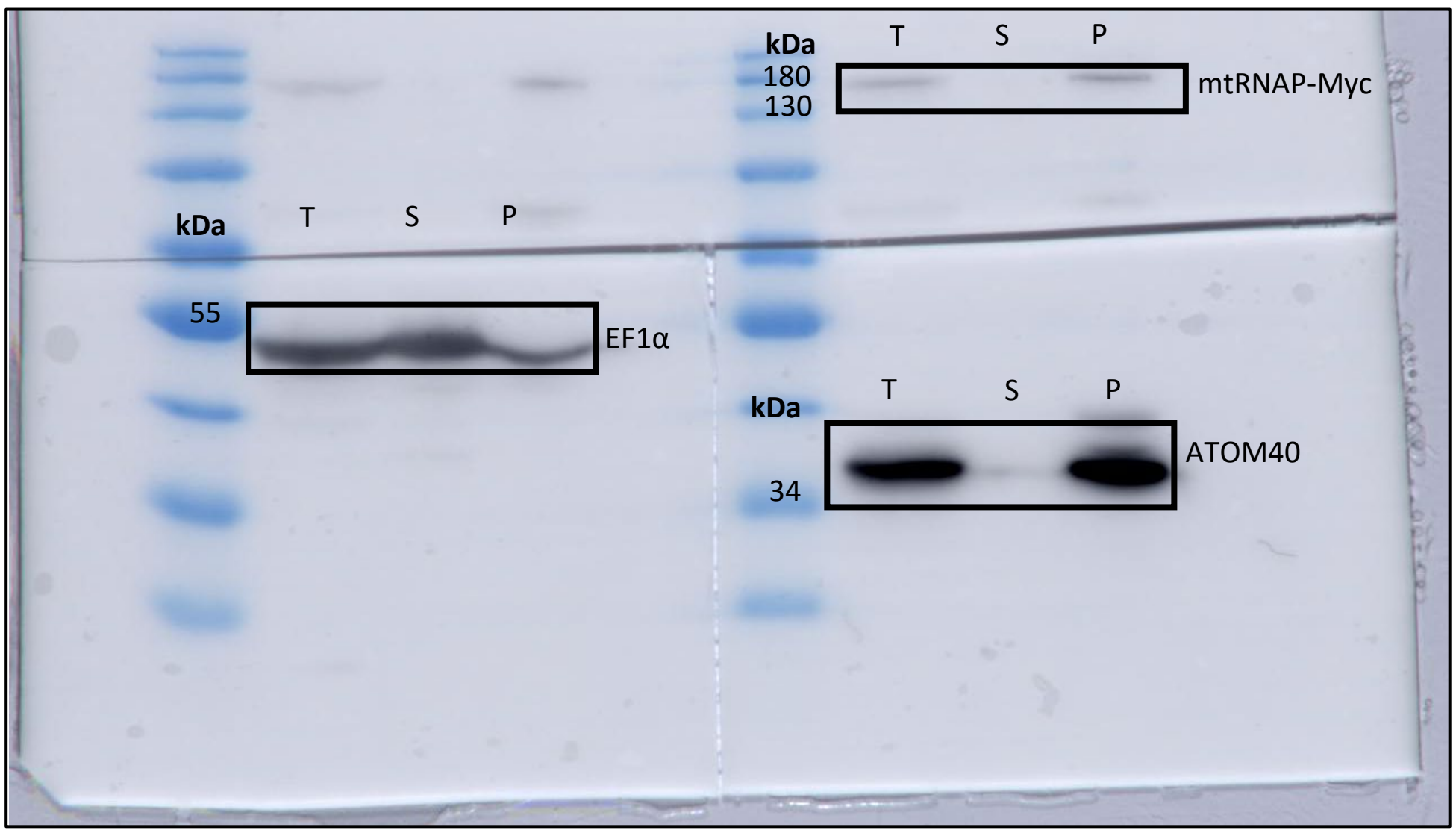

Figure 1: mtRNAP localizes to the kDNA and is essential in the procyclic form (PCF) and bloodstream form (BSF) of trypanosomes. (C)

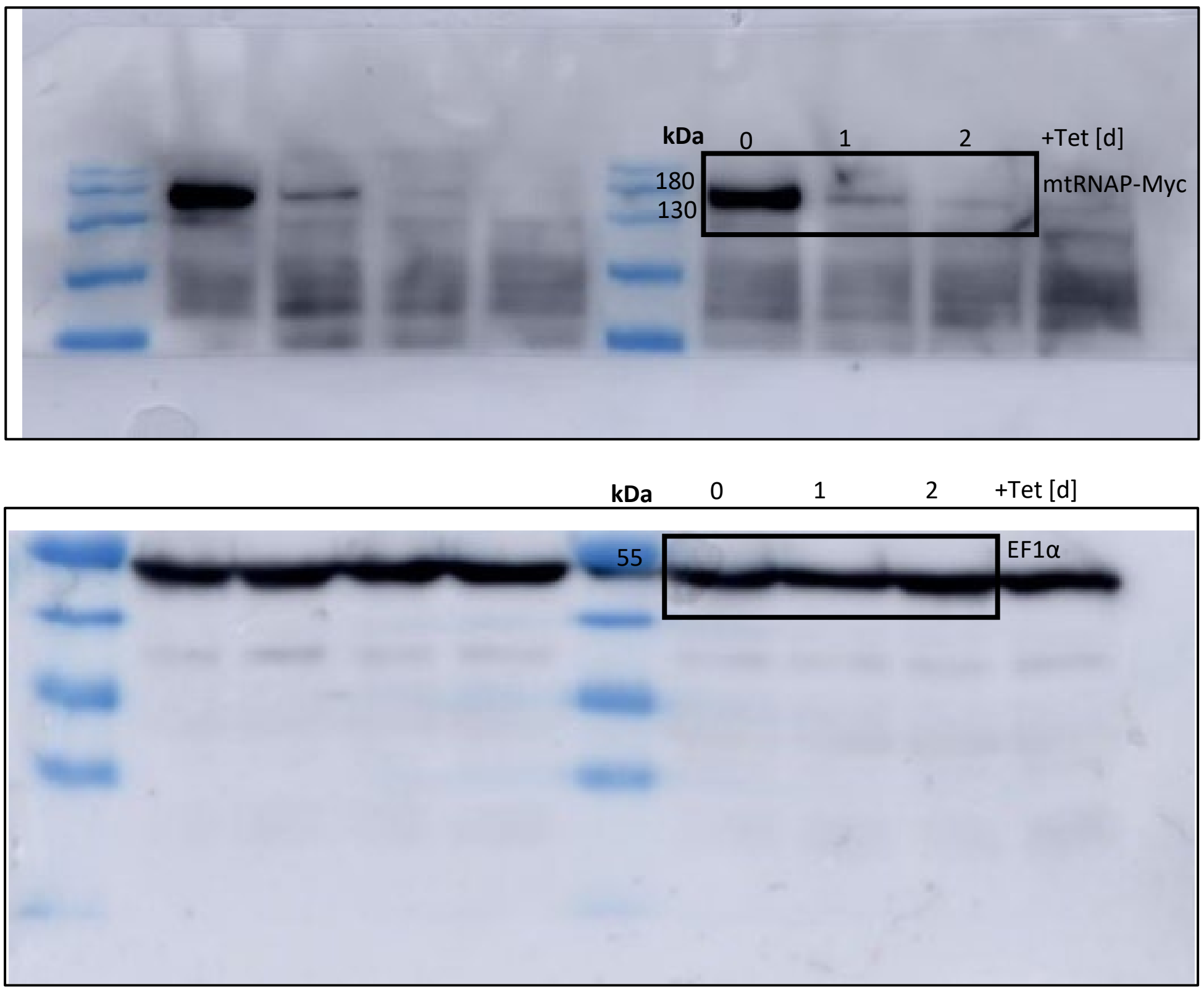

Figure 1: mtRNAP localizes to the kDNA and is essential in the procyclic form (PCF) and bloodstream form (BSF) of trypanosomes. (D) not that the blot containing mtRNAP-myc was cut and only this small portion could be retrieved

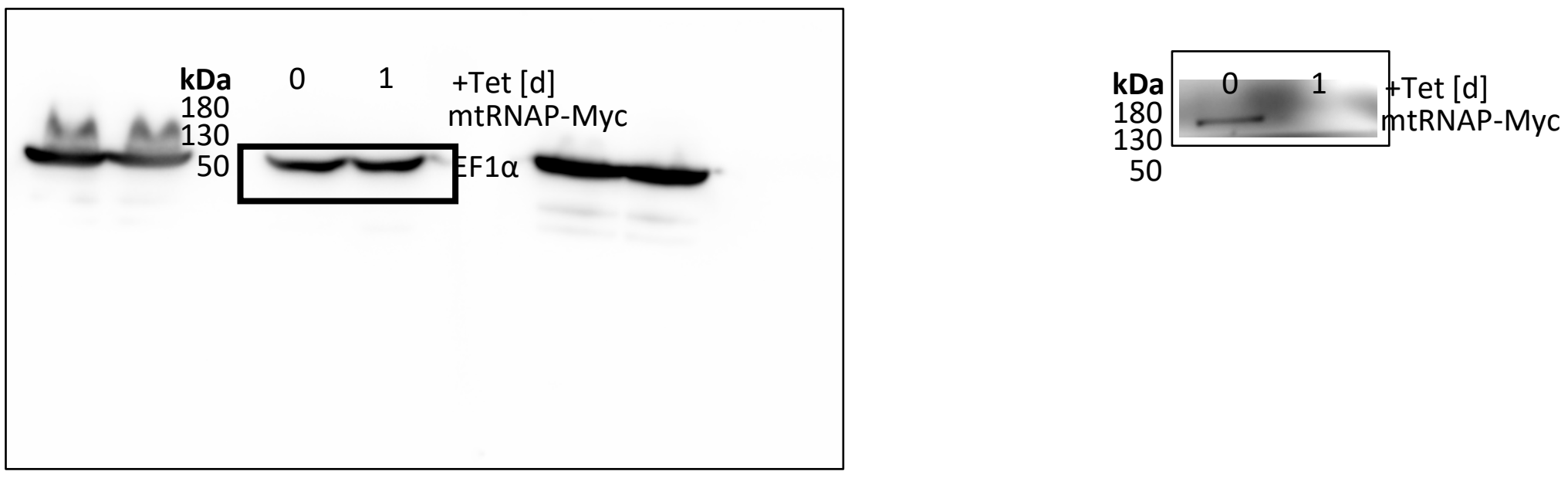

Figure 2: Immunoprecipitation analysis of mtRNAP to identify interaction partners. (A)

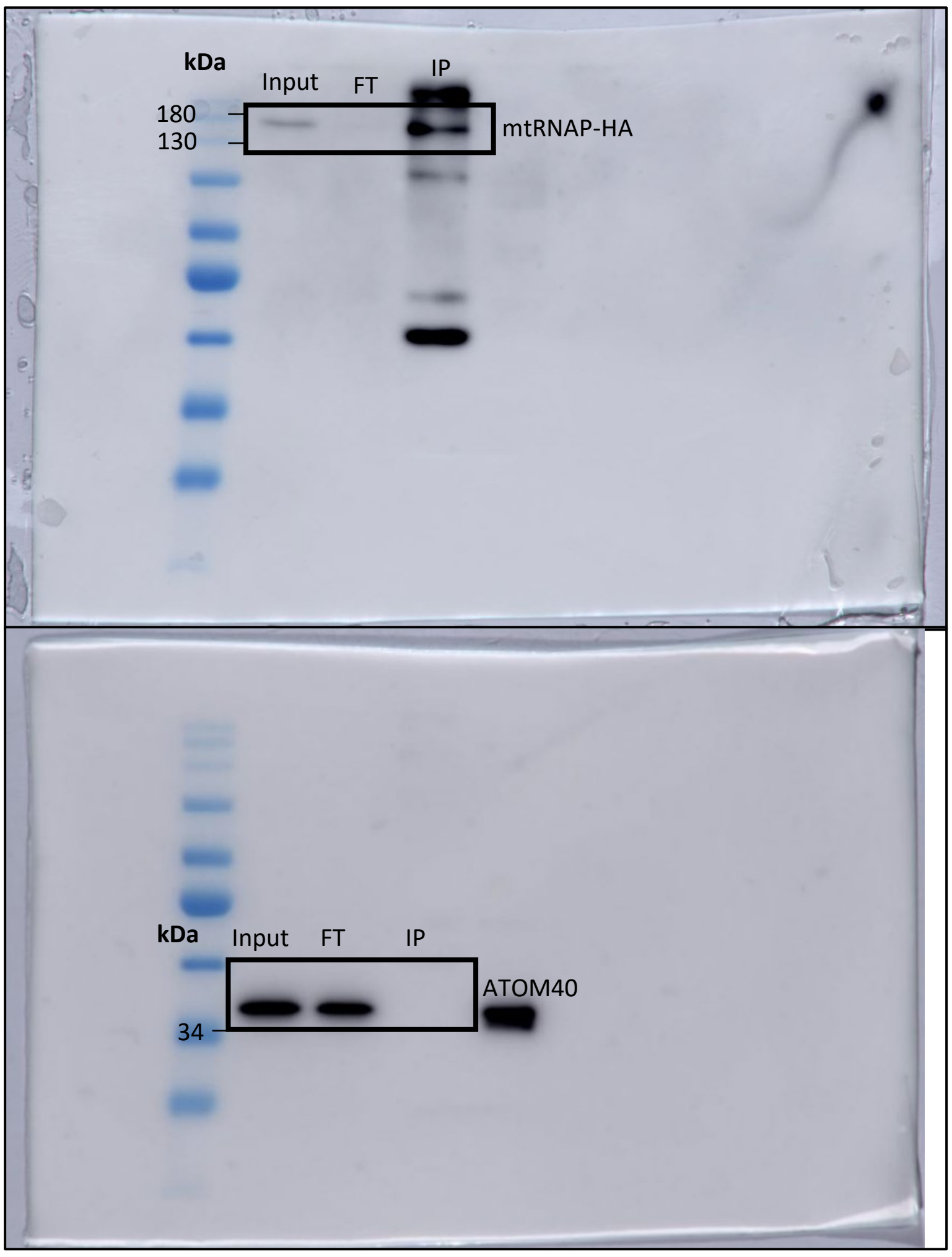

Figure 3: mtTF1 is conserved in the Kinetoplastea and co-localizes with mtRNAP at the kDNA in the PCF. (C and D)

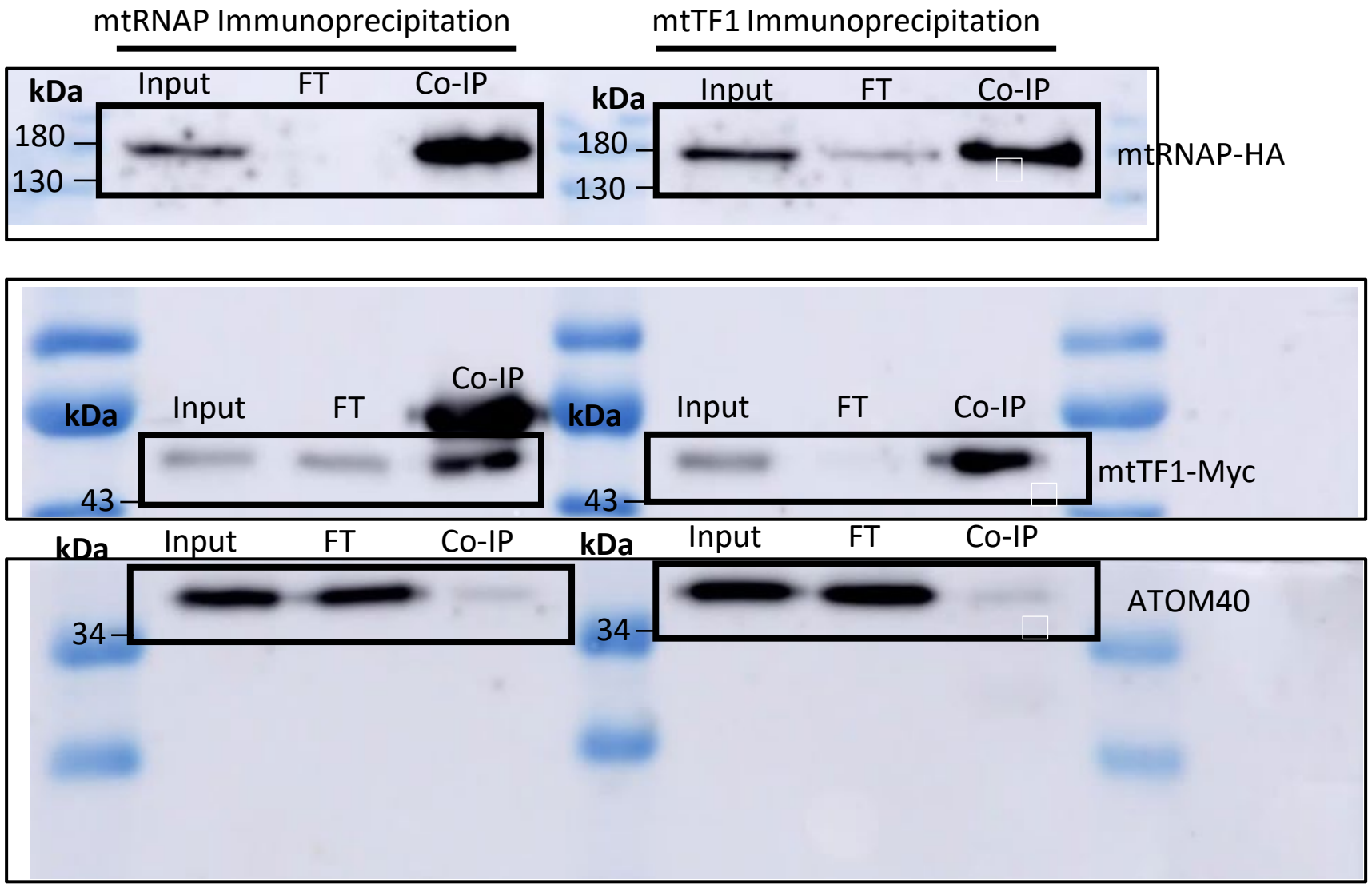

Figure 4: mtTF1 is essential in the insect form, and its localization at the kDNA depends on mtRNAP. (A)

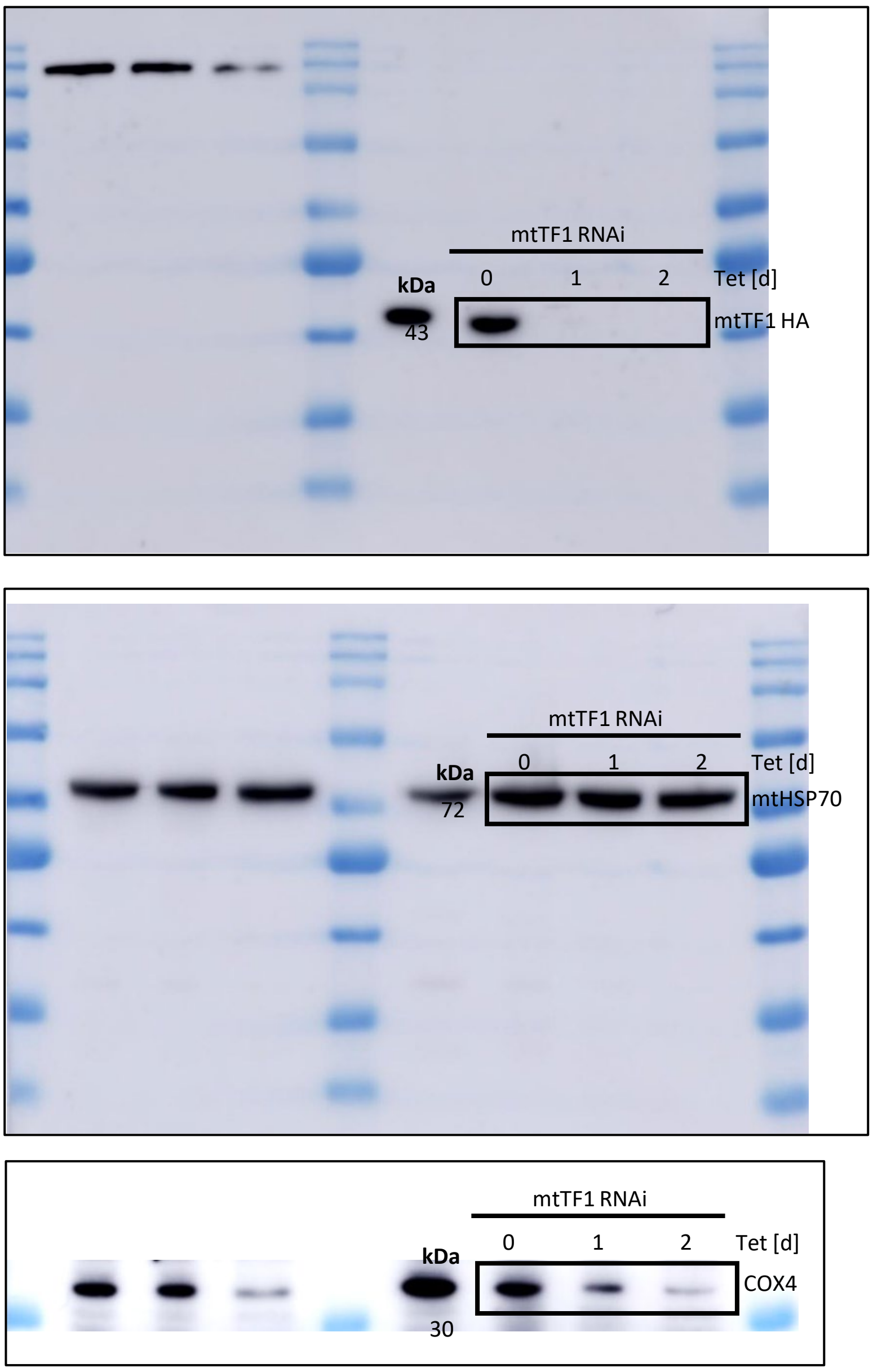

Figure 4: mtTF1 is essential in the insect form, and its localization at the kDNA depends on mtRNAP. (E)

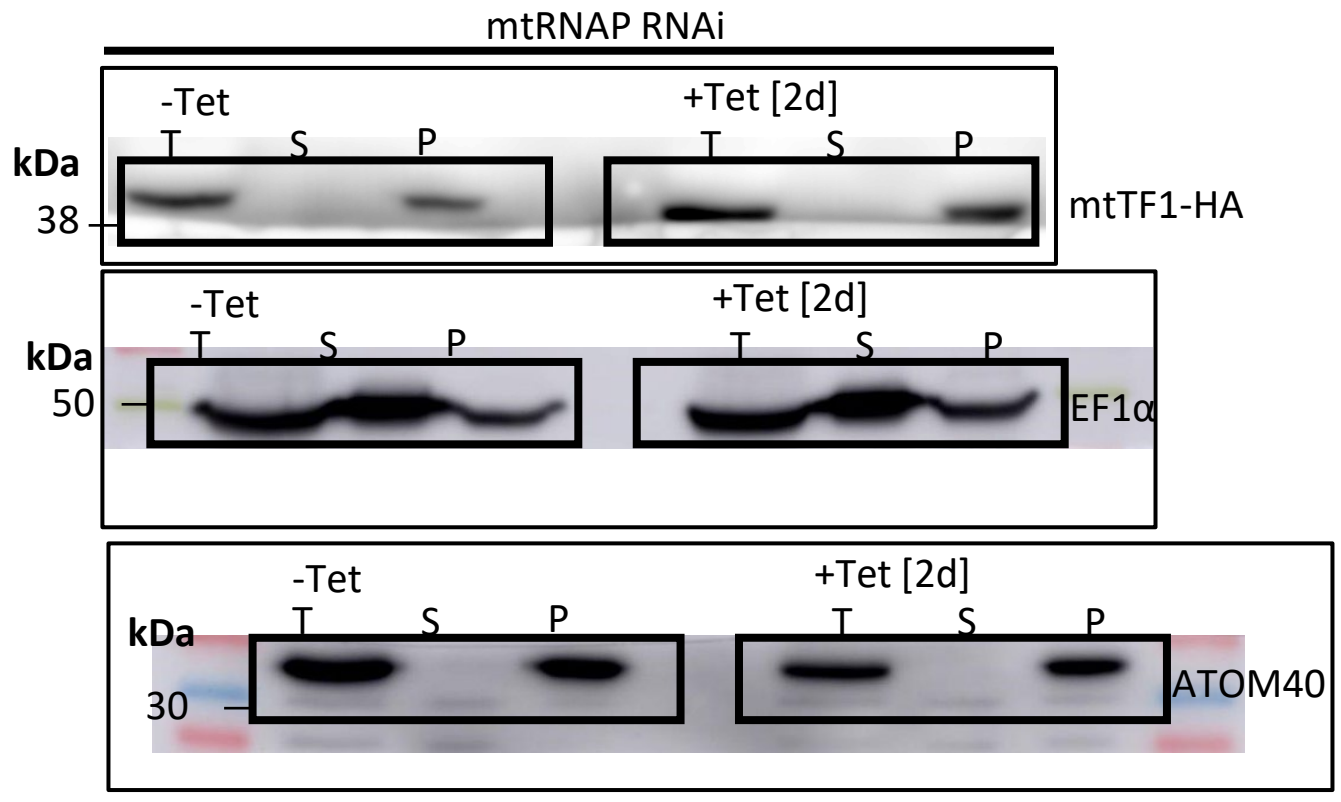

Figure 4: mtTF1 is essential in the insect form, and its localization at the kDNA depends on mtRNAP. (F)

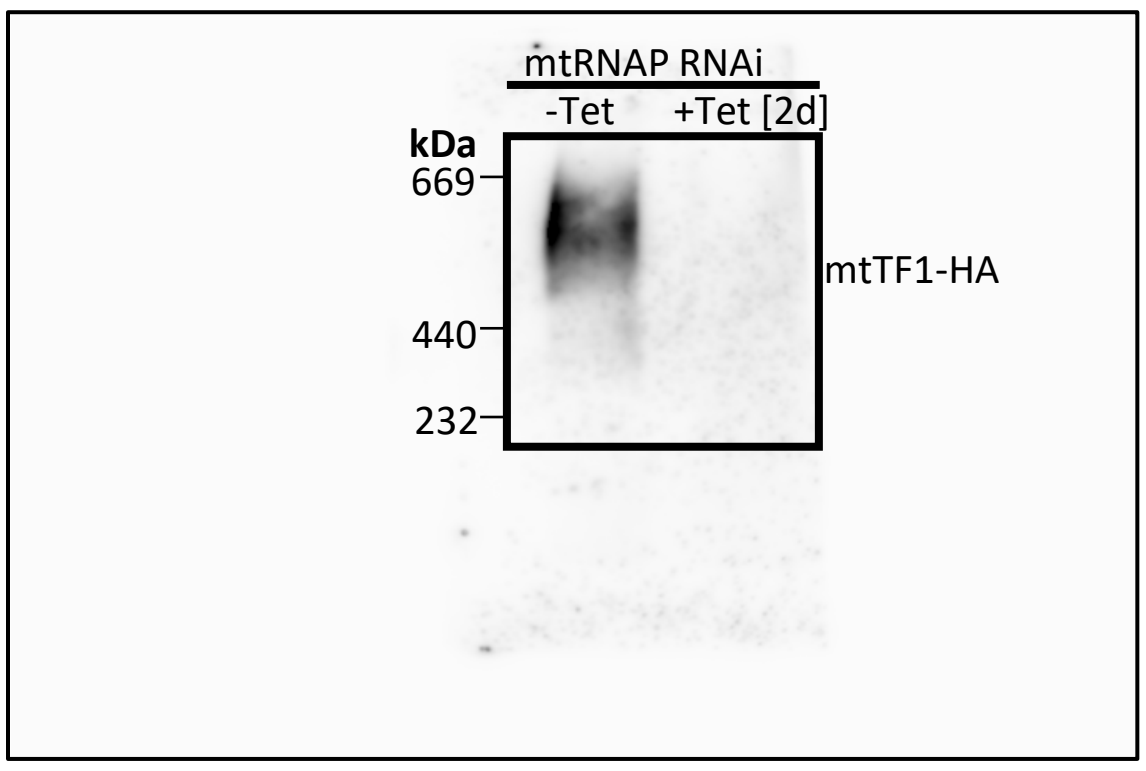

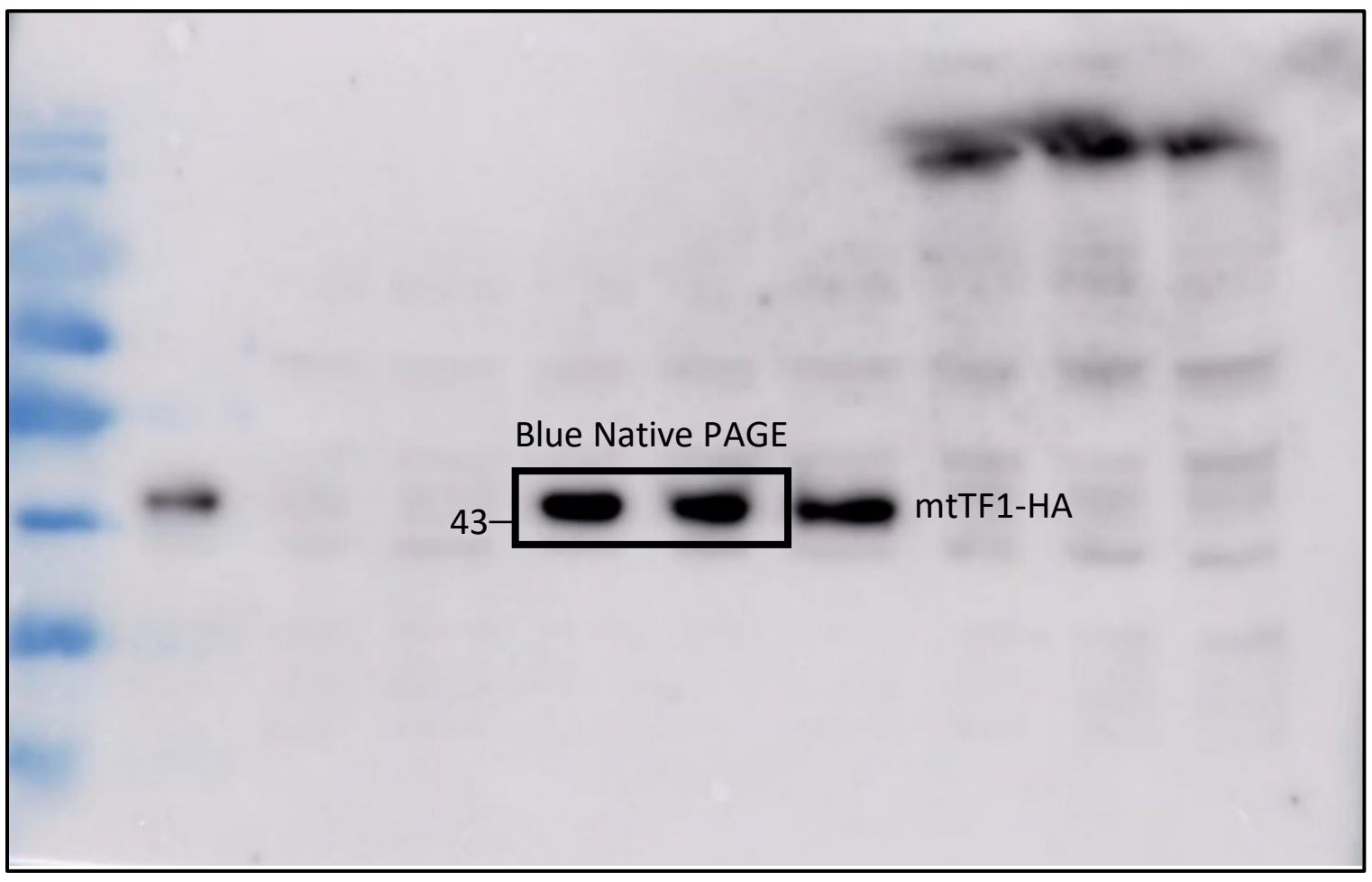

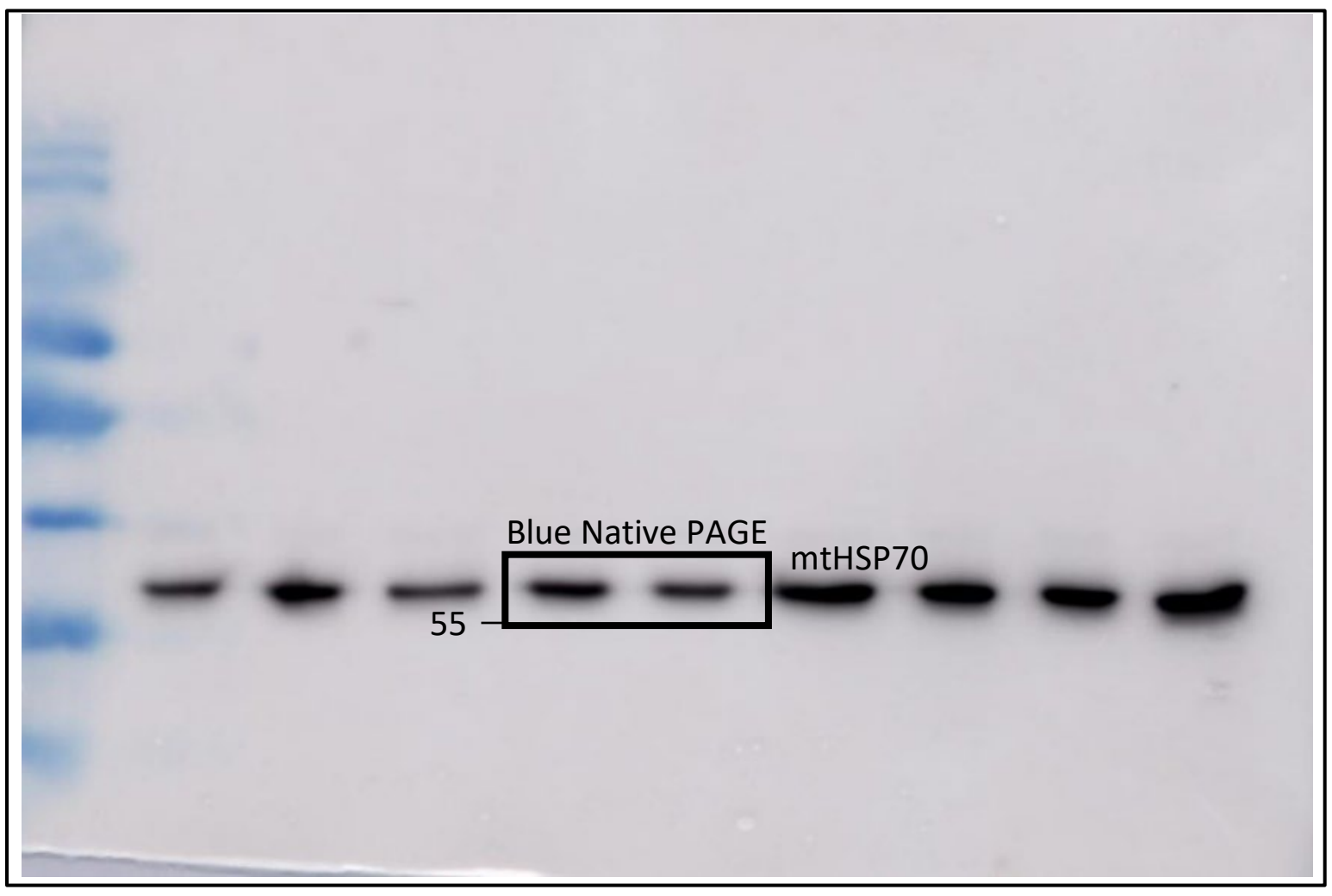

Figure 5: mtRNAP intensity at the kDNA decreases upon mtTF1 RNAi in the PCF. (D)

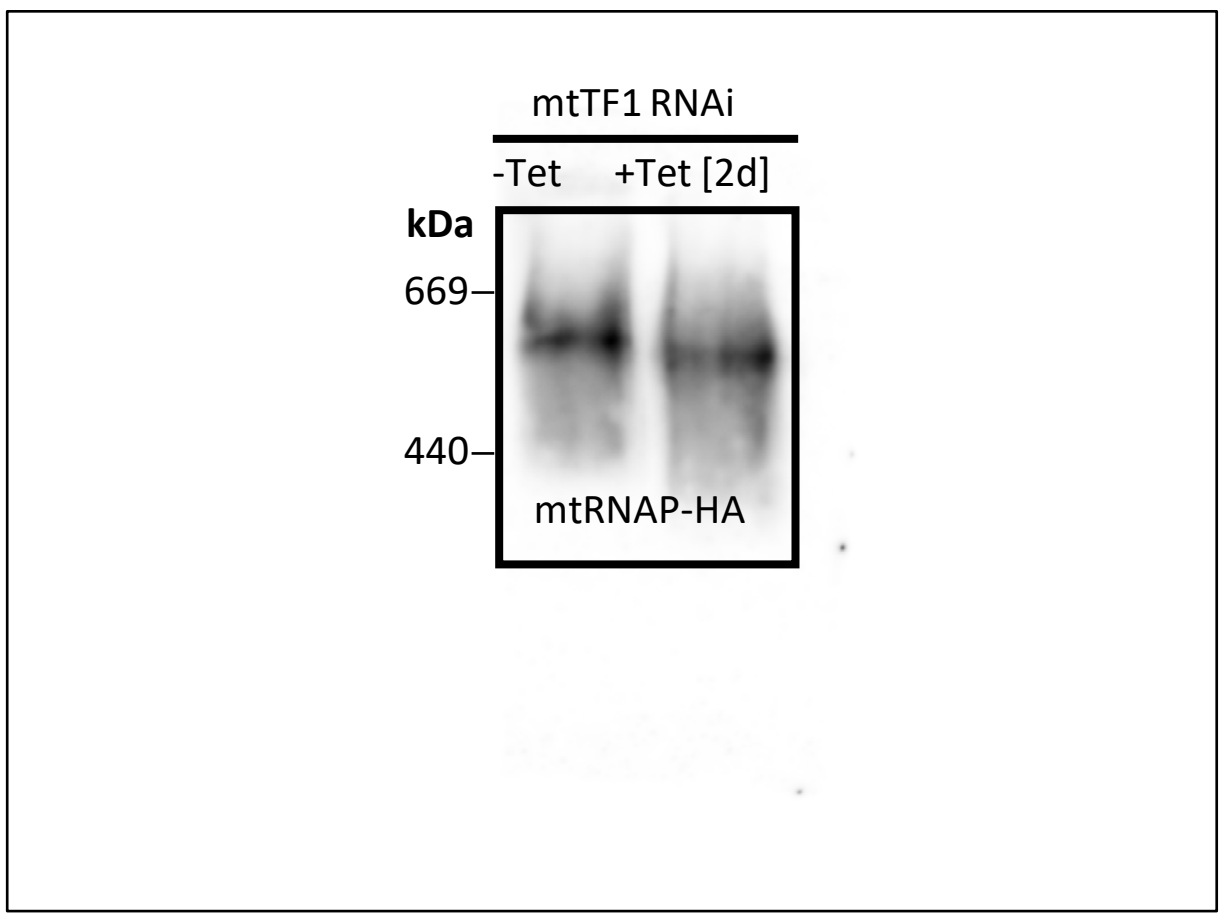

Figure 5: mtRNAP intensity at the kDNA decreases upon mtTF1 RNAi in the PCF. (E)

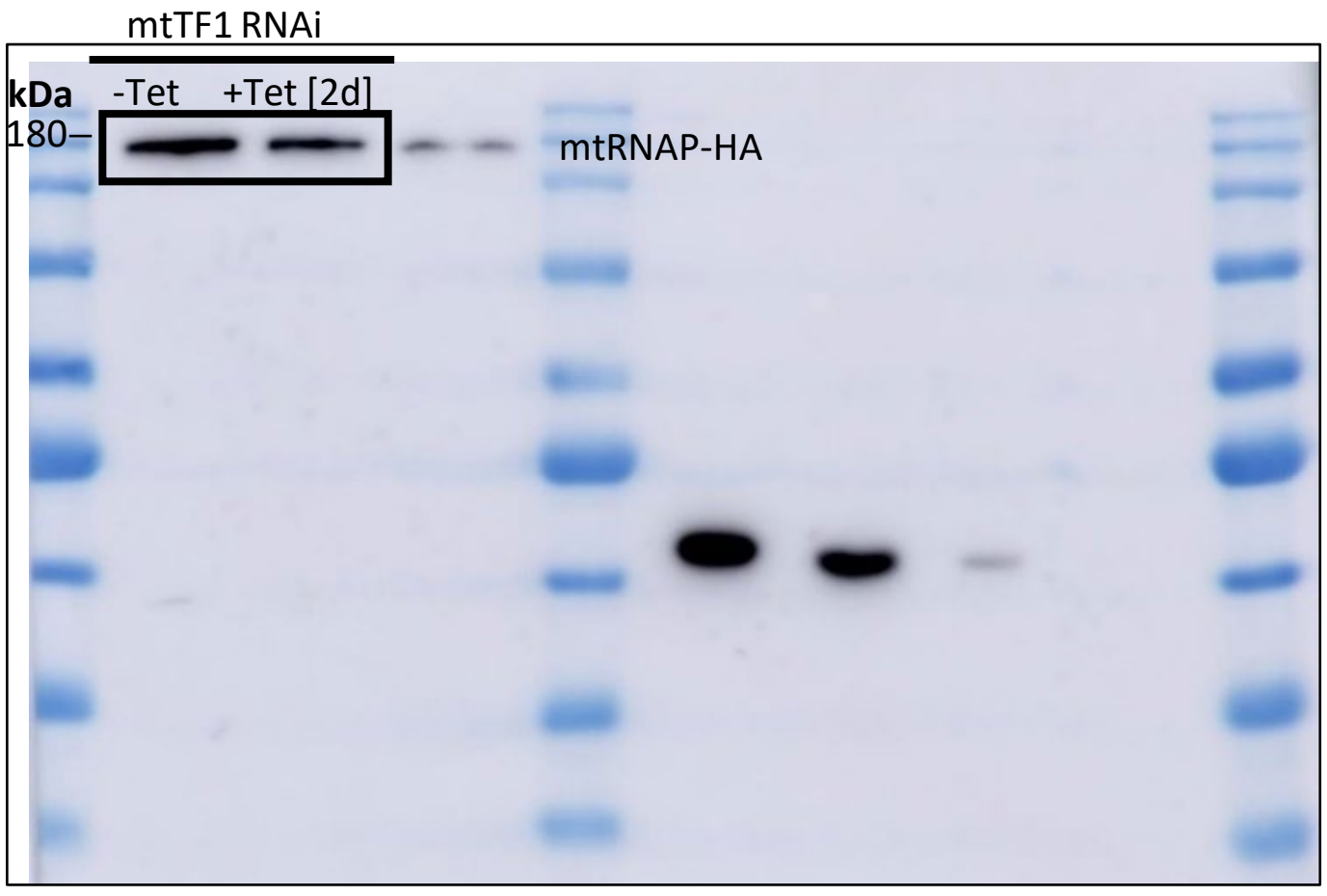

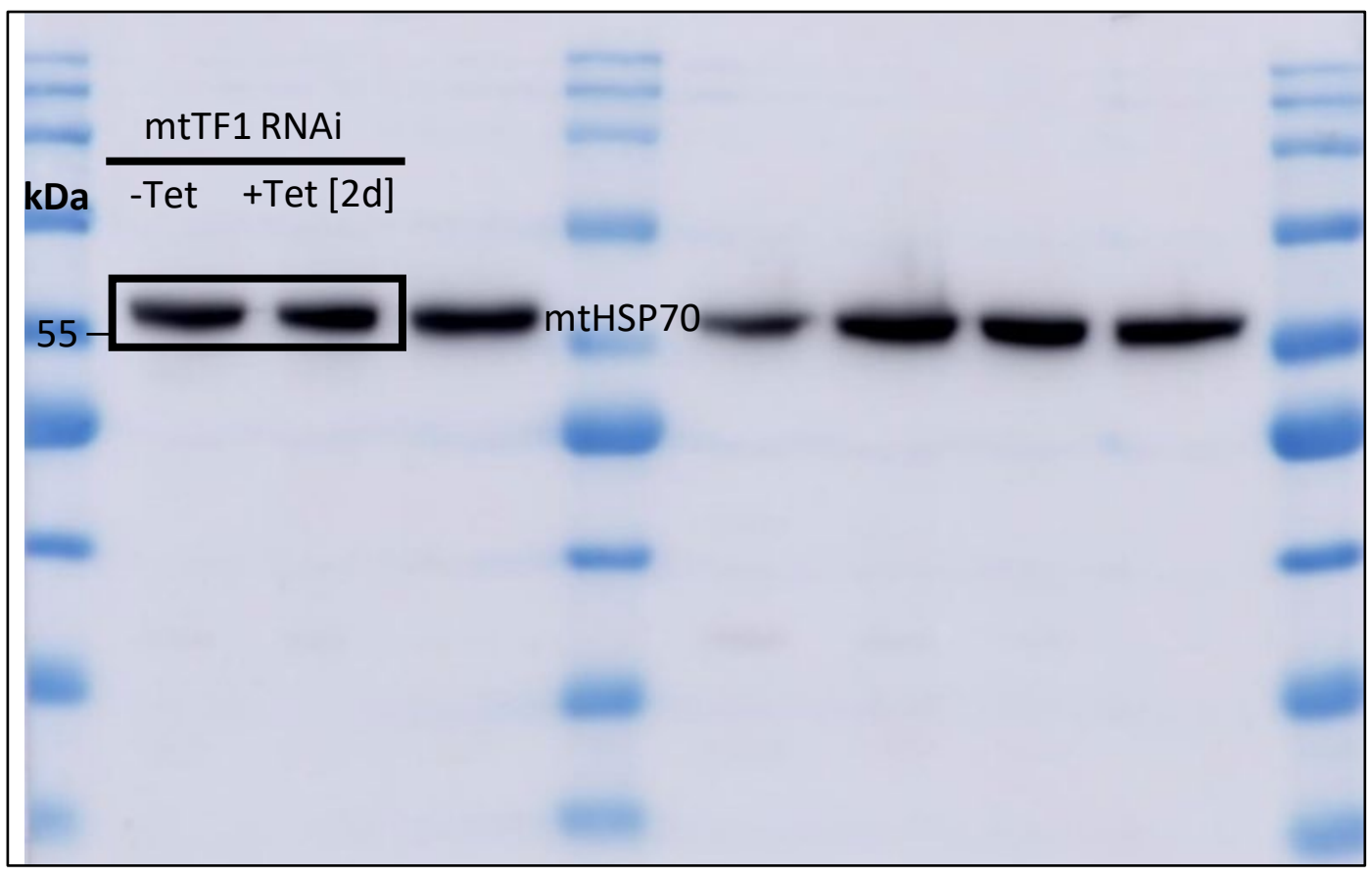

Figure 6: mtRNAP and mtTF1 are involved in maxicircle replication as well as maxi- and minicircle transcription in the PCF. (C and F)

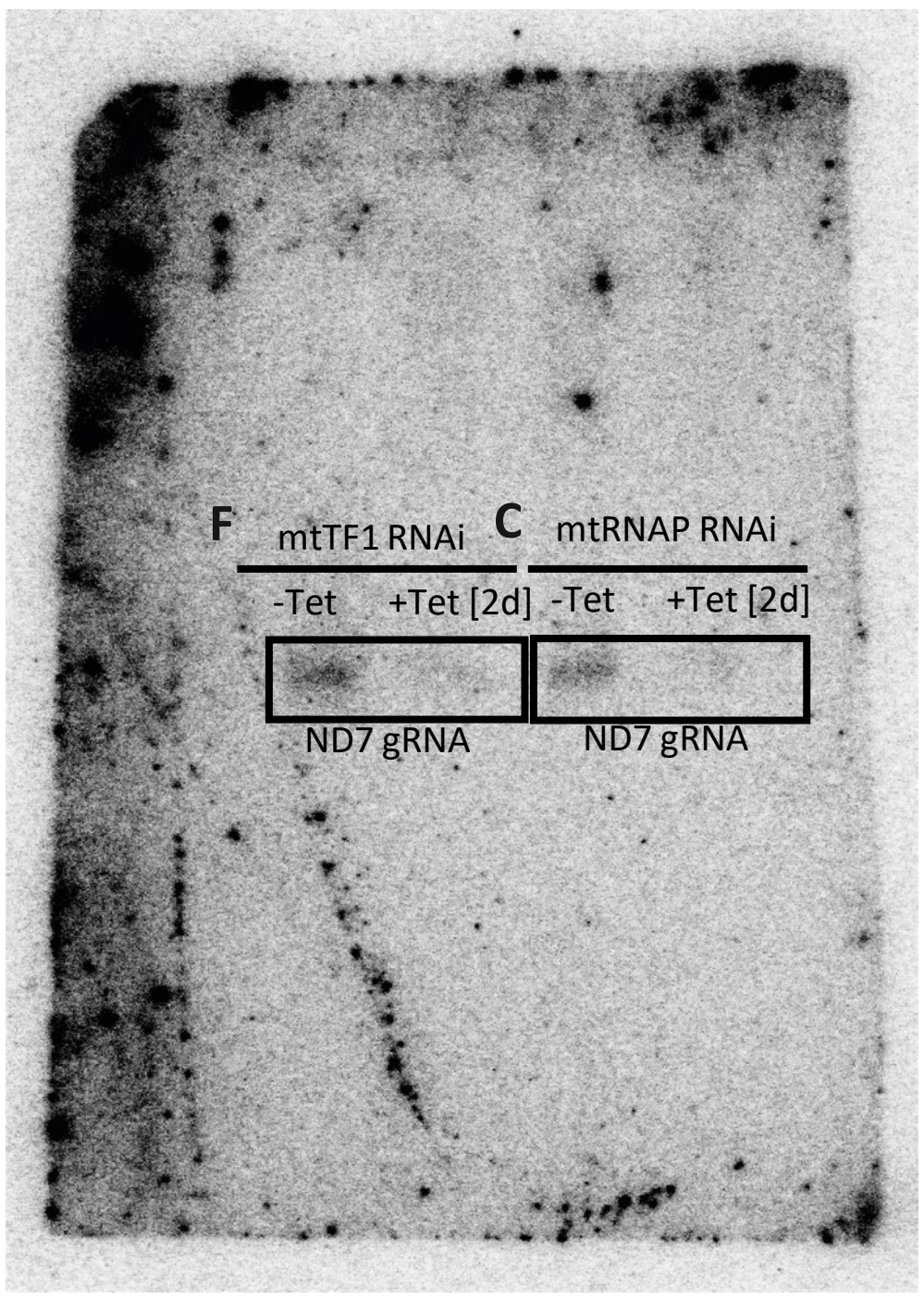

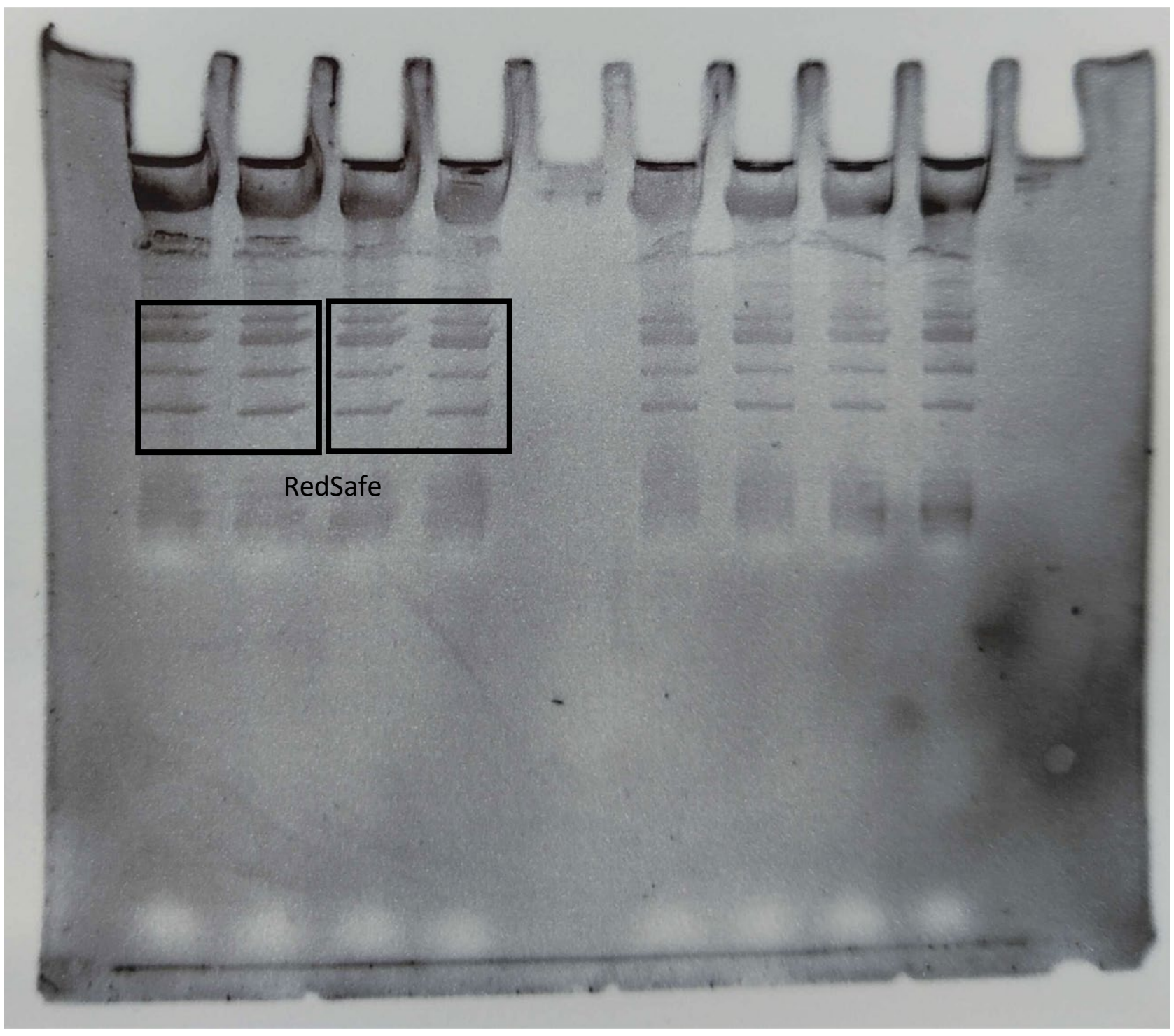

Supplementary Figure 1: mtRNAP-Myc expression does not rescue endogenous mtRNAP depletion by RNAi. the raw blots were accidentally deleted

Supplementary Figure 3: Immunoprecipitation analysis of mtTF1-myc verifies mtRNAP interaction. (A)

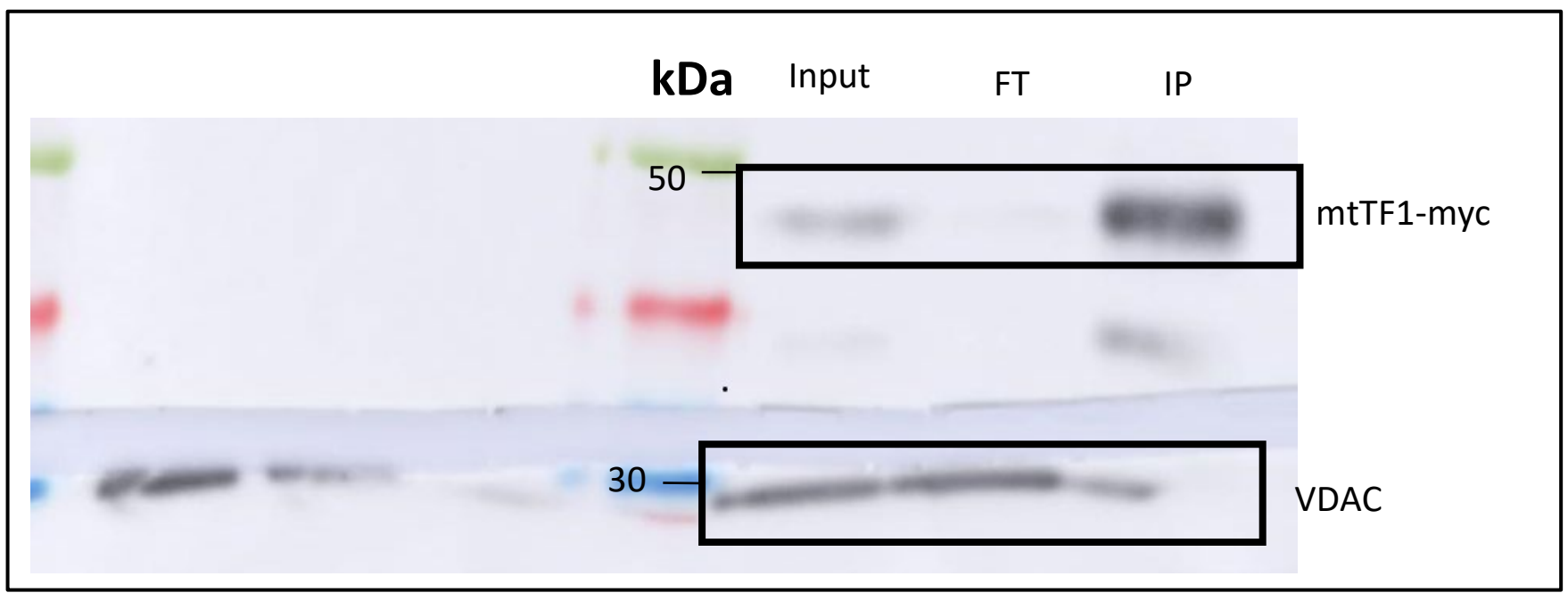

Supplementary Figure 4: Additional data for mtTF1-HA x mtRNAP RNAi and mtRNAP-HA x mtTF1 RNAi. (B)

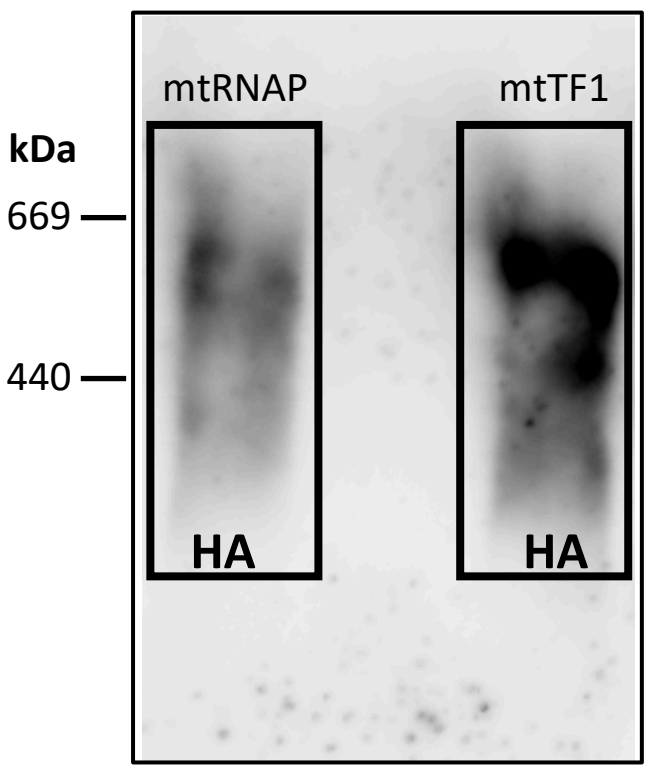

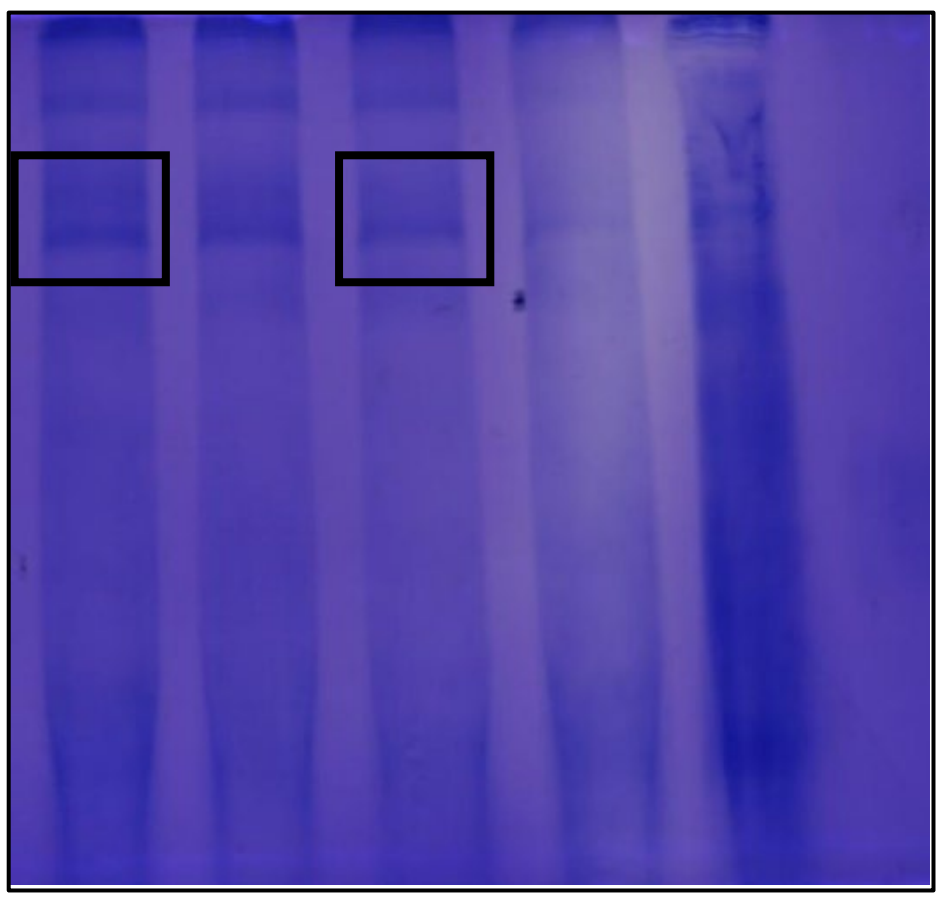

Supplementary Figure 4: Additional data for mtTF1-HA x mtRNAP RNAi and mtRNAP-HA x mtTF1 RNAi. (D)

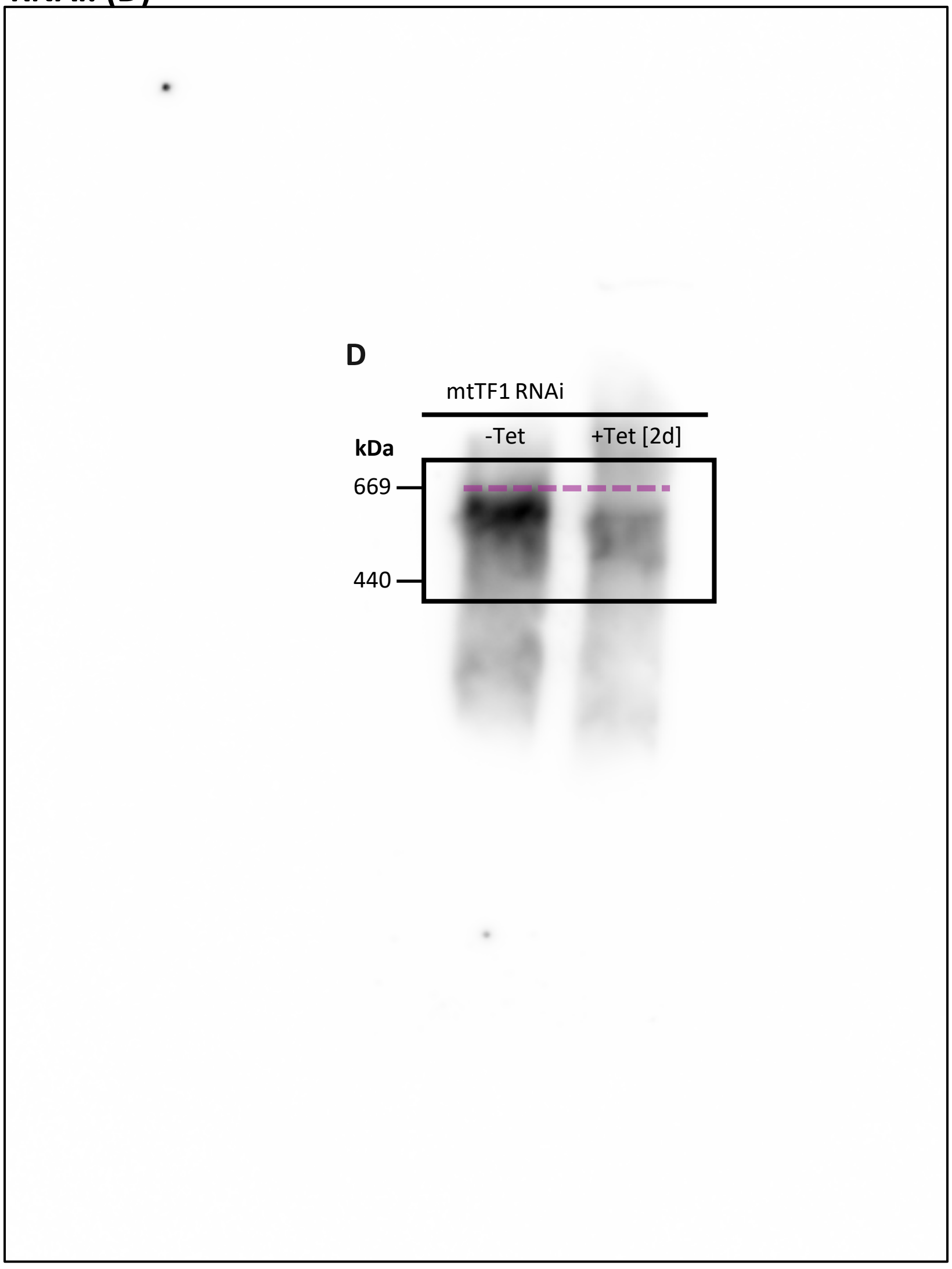

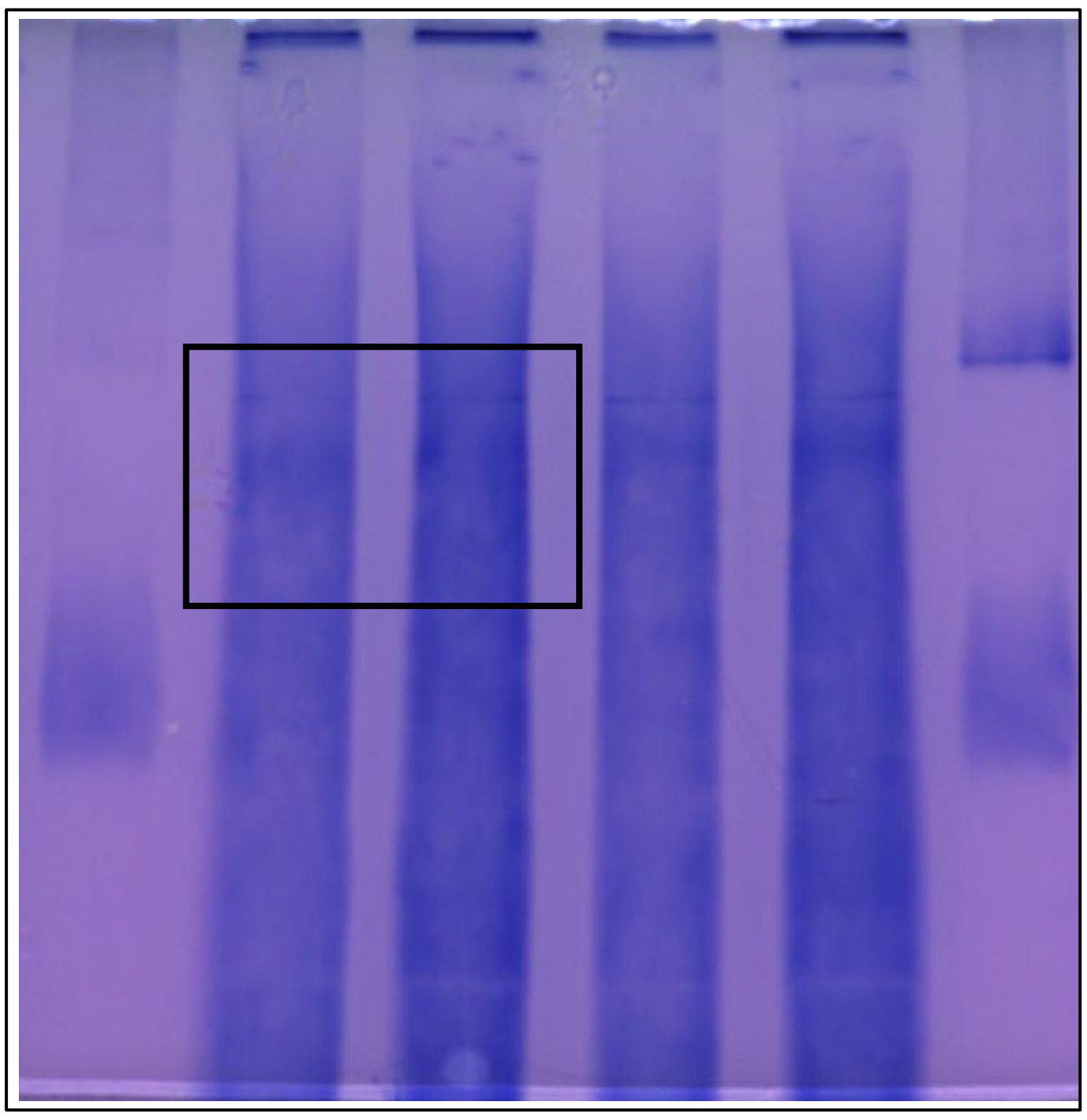

Supplementary Figure 4: Additional data for mtTF1-HA x mtRNAP RNAi and mtRNAP-HA x mtTF1 RNAi. (D)

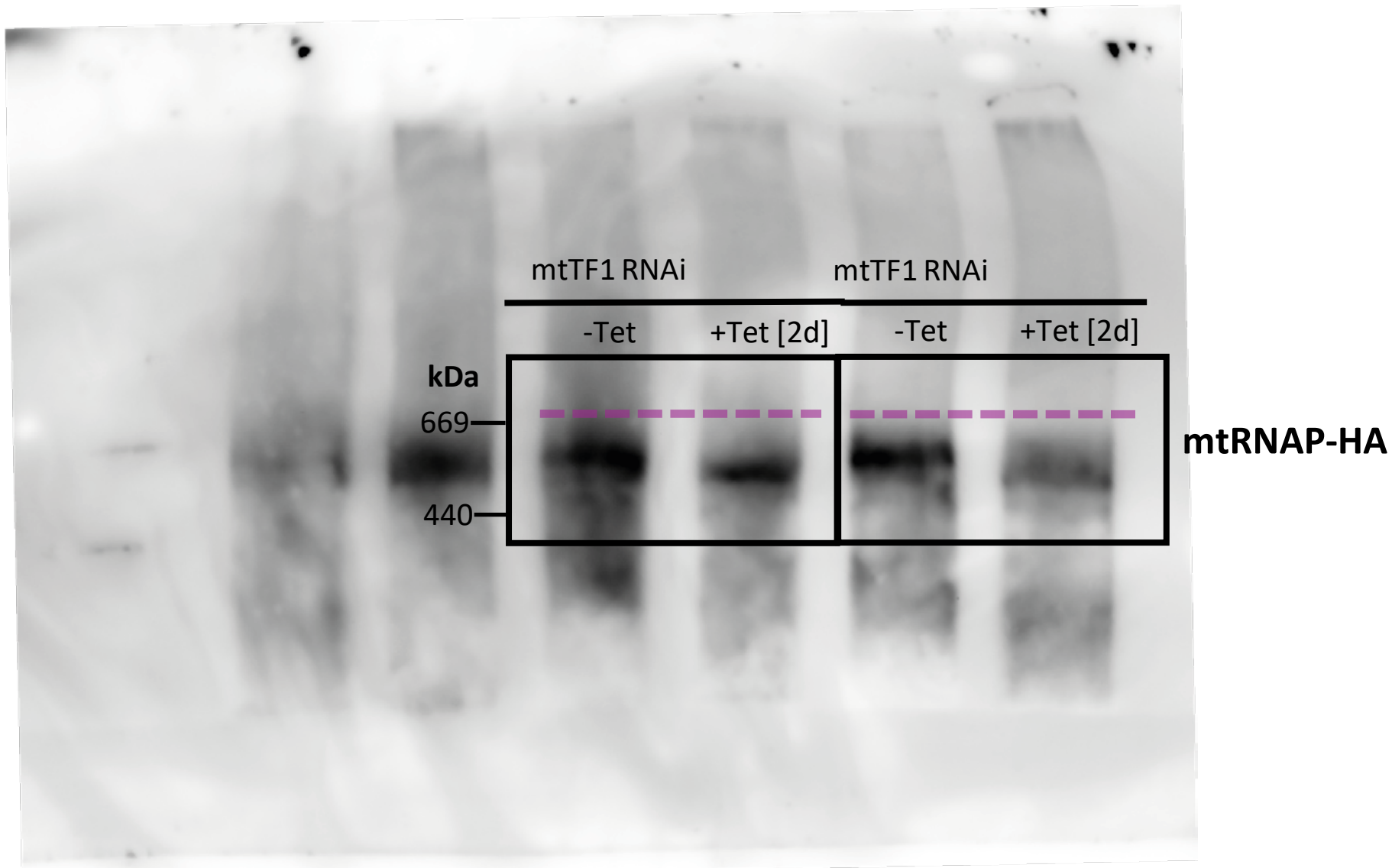

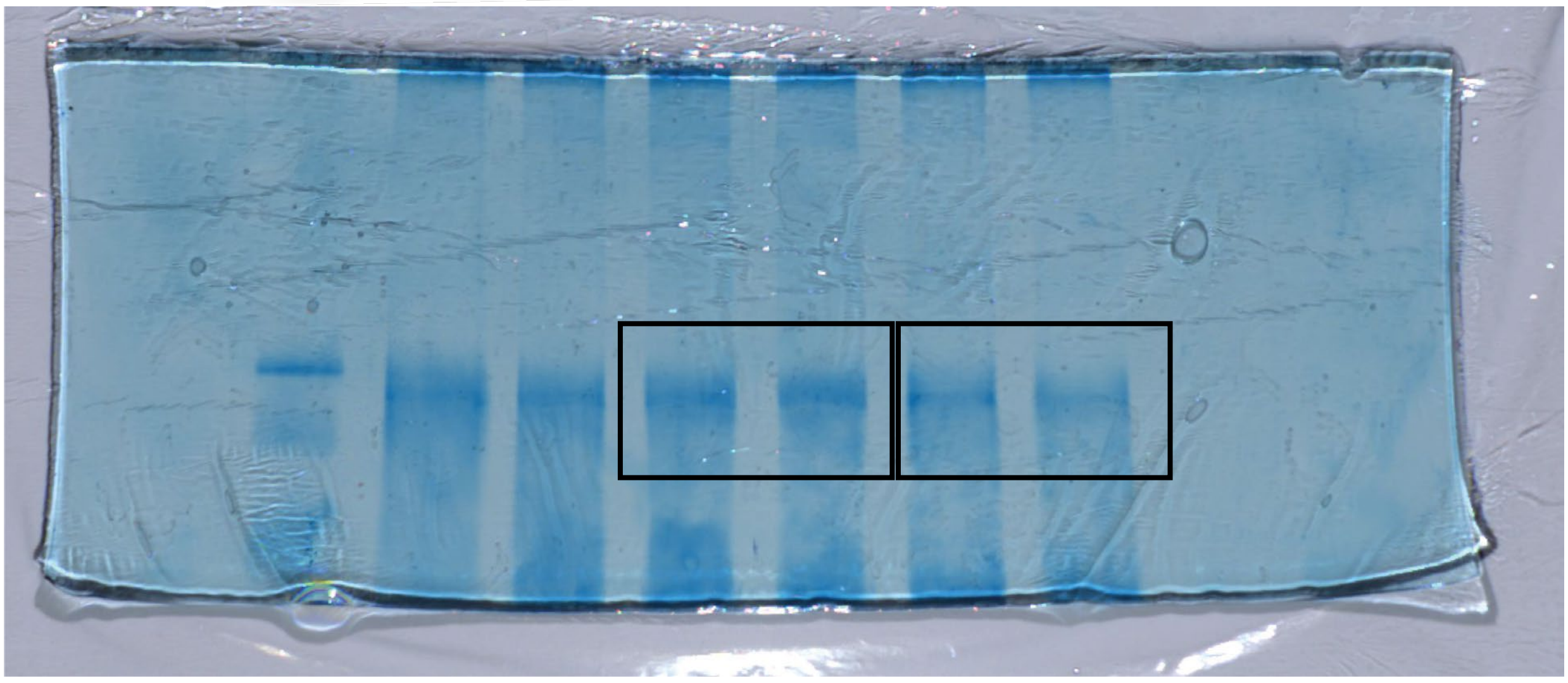

Supplementary Figure 6: Primase 1 localizes to the antipodal sites flanking the kDNA throughout the cell cycle and is essential for cell viability and kDNA replication in the PCF. (B)

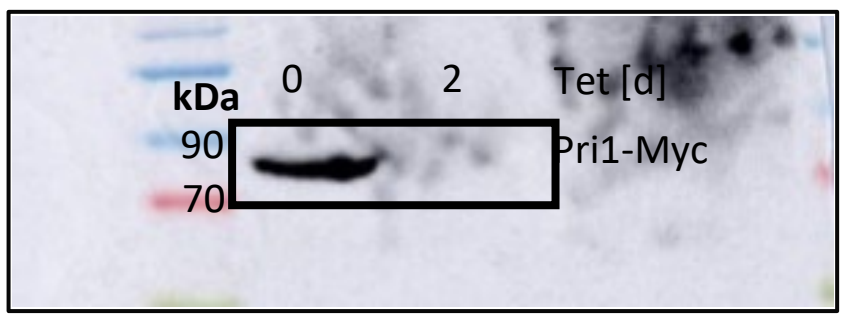

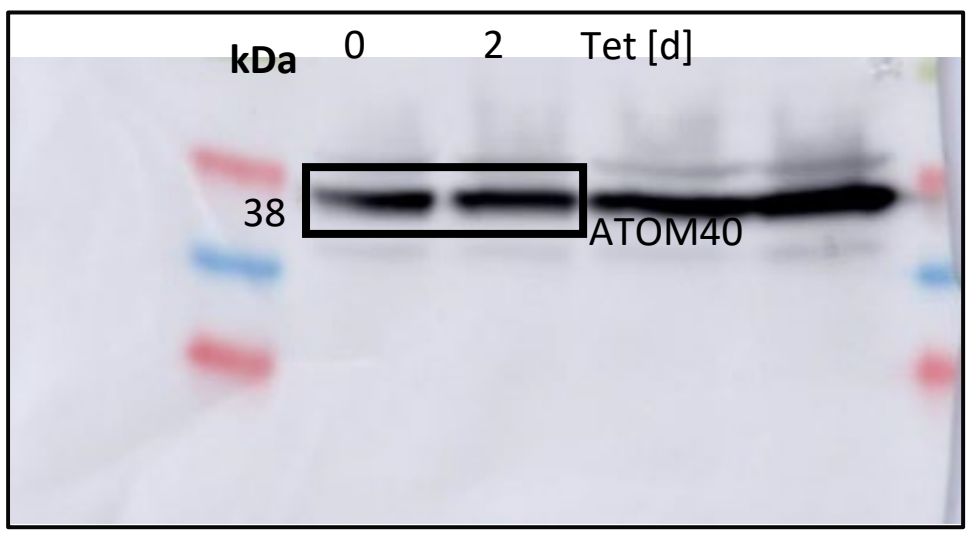

Supplementary Figure 7: mtTF1-strep expression rescues endogenous mtTF1 depletion by RNAi. the raw blots were accidentally deleted

Supplementary Figure 8: Purification of recombinant mtRNAP-mtTF1 complex. (A)

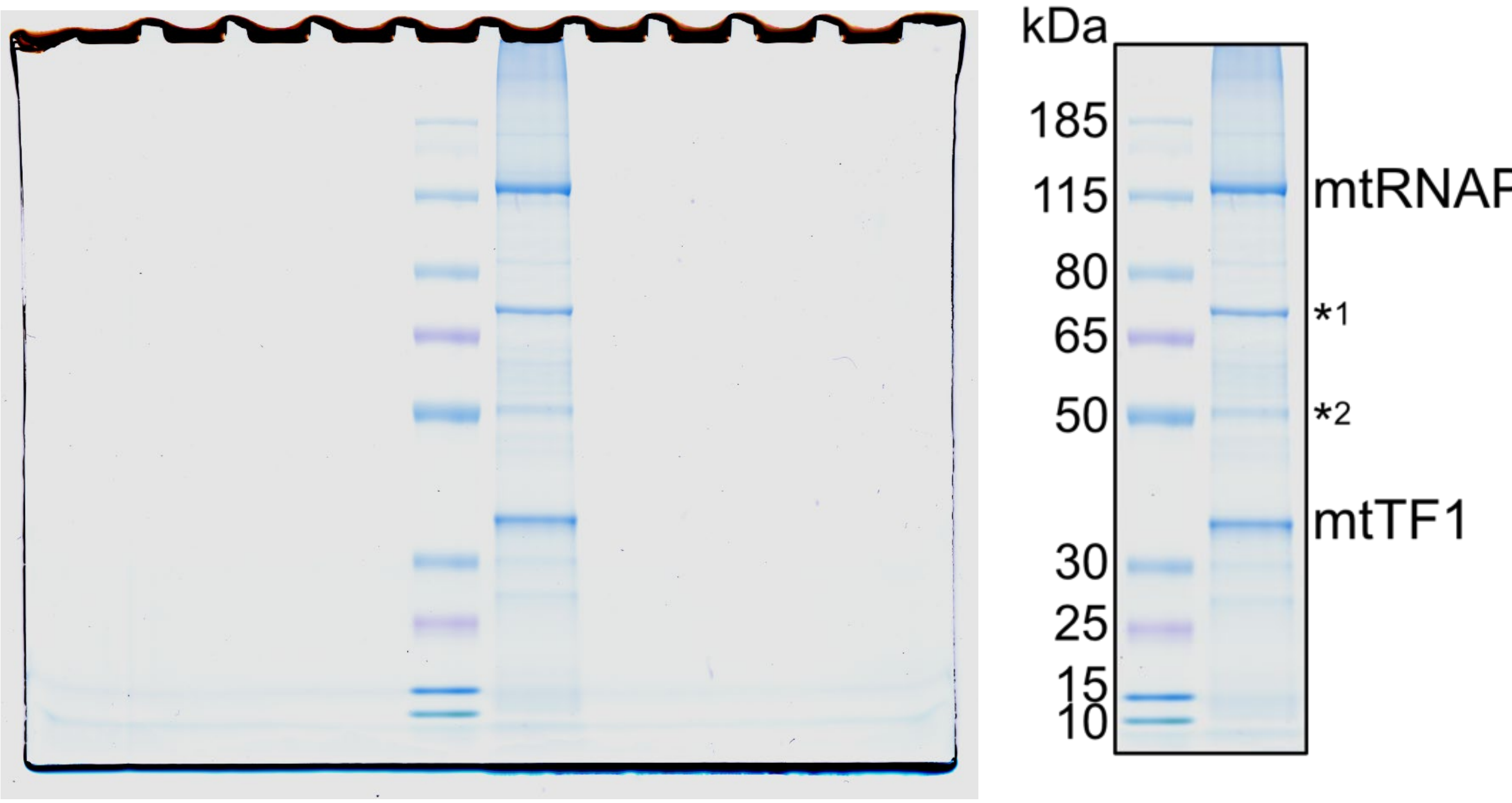

Supplement: Supplementary file 1 [file pbio.3003945.s009.pdf]
